# Differences in leaf and root litter decomposition in tropical montane rainforests are mediated by soil microorganisms not by decomposer microarthropods

**DOI:** 10.7717/peerj.14264

**Published:** 2022-11-03

**Authors:** Laura M. Sánchez-Galindo, Dorothee Sandmann, Franca Marian, Tobias Lauermann, Mark Maraun, Stefan Scheu

**Affiliations:** 1 JFB Institute of Zoology and Anthropology, University of Göttingen, Göttingen, Germany; 2 Centre of Biodiversity and Sustainable Land Use, University of Göttingen, Göttingen, Germany

**Keywords:** Acari, Collembola, Litterbags, Mesh size, Microbial biomass, Litter decomposition

## Abstract

**Background:**

Plant litter decomposition is a key process in carbon and nutrient cycling. Among the factors determining litter decomposition rates, the role of soil biota in the decomposition of different plant litter types and its modification by variations in climatic conditions is not well understood.

**Methods:**

In this study, we used litterbags with different mesh sizes (45 µm, 1 mm and 4 mm) to investigate the effect of microorganisms and decomposer microarthropods on leaf and root litter decomposition along an altitudinal gradient of tropical montane rainforests in Ecuador. We examined decomposition rates, litter C and N concentrations, microbial biomass and activity, as well as decomposer microarthropod abundance over one year of exposure at three different altitudes (1,000, 2,000 and 3,000 m).

**Results:**

Leaf litter mass loss did not differ between the 1,000 and 2,000 m sites, while root litter mass loss decreased with increasing altitude. Changes in microbial biomass and activity paralleled the changes in litter decomposition rates. Access of microarthropods to litterbags only increased root litter mass loss significantly at 3,000 m. The results suggest that the impacts of climatic conditions differentially affect the decomposition of leaf and root litter, and these modifications are modulated by the quality of the local litter material. The findings also highlight litter quality as the dominant force structuring detritivore communities. Overall, the results support the view that microorganisms mostly drive decomposition processes in tropical montane rainforests with soil microarthropods playing a more important role in decomposing low-quality litter material.

## Introduction

Dead leaves and roots comprise the major plant litter material that enters the belowground system and represent the main energy resource for soil organisms (Berg & McClaugherty, 2008). Although the annual input of leaf and root litter in forests is typically equivalent in mass (Norby et al., 2004; Freschet et al., 2013), most studies investigating the effects of soil organisms on plant litter decay focus on leaves, overlooking the potential of roots as a food resource and regulator of carbon and nutrient cycling (García-Palacios et al., 2016; Fujii & Takeda, 2017). Therefore, integration of both root litter and leaf litter is needed for a comprehensive understanding of the role of soil organisms in element cycling and ecosystem functioning.

Leaf and root litter differ in structure and chemical composition (Berg & McClaugherty, 2008). Generally, roots contain higher concentrations of recalcitrant compounds compared to leaves, which inhibits degradation by soil organisms and this is assumed to be the reason for the slower decay rates of roots compared to leaves (Hobbie et al., 2010; Freschet, Aerts & Cornelissen, 2012; García-Palacios et al., 2016; Jo, Fridley & Frank, 2016). Additionally, leaf and root litter are found at different positions on the forest floor. Leaf litter is deposited on top of the soil, while root litter enters the decomposer system directly within the soil. These different locations generate different input pathways of nutrients and are associated with varying microenvironments for soil organisms (Ostertag & Hobbie, 1999; Fujii & Takeda, 2010; Fujii & Takeda, 2017). Differences in quality and input pathways of litter types are likely to affect the abundance, composition and activity of soil organisms, with knock-on effects for decomposition rates and soil nutrient dynamics.

Effects of soil organisms on litter decomposition not only change with litter traits, but also with climatic conditions (Coûteaux, Bottner & Berg, 1995; Aerts, 1997; Hättenschwiler, Tiunov & Scheu, 2005). In tropical Andean montane ecosystems climatic conditions change strongly with altitude (Myers et al., 2000; Beck & Ritcher, 2008). Increasing altitude is associated with a decline in litter nutrient concentrations and an increase in the thickness of soil organic layers and fine root biomass (diameter < 2 mm) (Tanner, Vltousek & Cuevas, 1998; Soethe, Lehmann & Engels, 2007; Graefe, Hertel & Leuschner, 2008). Notably, at higher altitudes, more organic material originates from fine root litter than from fallen leaves (Röderstein, Hertel & Leuschner, 2005). Such changes in litter traits with environmental conditions affect the nutrient supply for decomposer organisms and thereby likely control the abundance and diversity of soil decomposer species (Wang, Ruan & Han, 2010; García-Palacios et al., 2016). Variations in climatic conditions may also induce changes in soil community composition resulting in well-adapted communities able to govern decomposition processes (Allison et al., 2013). However, few studies have investigated the impacts of litter traits and altitudinal changes in climate on soil animal communities and their role in leaf and root litter decomposition (Decaëns et al., 2006; Bradford et al., 2017), particularly in tropical montane rainforest ecosystems (Marian et al., 2017; Marian et al., 2018).

Decomposer communities in tropical montane rainforests are dominated by microorganisms and soil microarthropods, whereas large decomposer species are virtually lacking (Illig et al., 2008; Maraun et al., 2008; Scheu et al., 2008). Among soil microarthropods, oribatid mites (Oribatida, Acari) and springtails (Collembola) are the most abundant and diverse worldwide (Seastedt, 1984; Ruess & Lussenhop, 2005). These microarthropods increase litter decomposition rates and nutrient cycling in forest ecosystems either *via* litter consumption or through stimulation of microbial activity and transport of microbial propagules (Swift, Heal & Anderson, 1979; Seastedt, 1984; Ruess & Lussenhop, 2005). However, the positive effect of decomposer microarthropods on microbial growth has been reported to be density-dependent and restricted to low density (Kaneda & Kaneko, 2008; Crowther & A’Bear, 2012; Crowther, Boddy & Jones, 2012; Gergócs & Hufnagel, 2016). In tropical montane rainforests, the density of microarthropods is low compared to *e.g.*, temperate forests, suggesting that they may stimulate or little affect microorganisms and decomposition processes (Illig et al., 2008; Marian et al., 2017; Sánchez-Galindo et al., 2021). Nevertheless, the role of microarthropods in decomposition processes is likely to vary with altitude and litter types, but this has been little studied.

The present study investigates the effects of microorganisms and decomposer microarthropods on leaf and root litter decomposition and their variations along an altitudinal gradient of tropical montane rainforests in Ecuador. Decomposition rates, microbial biomass and respiration, as well as Collembola and Oribatida abundances and Oribatida community composition, were studied over one year using litterbags with different mesh sizes to control access by soil fauna to the litter. We hypothesized that (1) the decomposition of litter decreases with increasing altitude, and this is mainly due to less favourable abiotic conditions. We also hypothesized that (2) access of litter by soil microarthropods increases decomposition as well as microbial biomass and activity in both leaf and root litter. Further, we hypothesized that (3) the abundance of decomposer microarthropods and Oribatida species richness increase with increasing microbial biomass in both leaf and root litter. Lastly, we hypothesized that (4) increase in litter quality (as indicated by litter C-to-N ratio) and microbial biomass during decomposition are key factors structuring Oribatida communities in both leaf and root litter.

## Materials & Methods

### Study area

The study area is in the northern fringes of the Podocarpus National Park in the eastern slopes of the Andean Cordillera, Southeast Ecuador. Three study sites, situated at 1,000, 2,000 and 3,000 m a.s.l. were selected to represent an altitudinal gradient with moderately steep slopes of 26–31° (Moser, Hertel & Leuschner, 2007). The lower site at 1,000 m a.s.l. (S04 °06′54″, W78 °58′02″) is in the Río Bombuscaro valley and classified as evergreen submontane rainforest dominated by tree species of Arecaceae, Combretaceae, Moraceae, Monimiaceae, Rubiaceae and Sapotaceae (Homeier et al., 2008). The intermediate site at 2,000 m a.s.l. (S3 °58′18″, W79 °4′45″) is in the Reserva Biológica San Francisco on the north-facing flank of the Río San Francisco valley and consists of an evergreen lower montane rainforest dominated by trees of Arecaceae, Clusiaceae, Ericaceae, Lauraceae, Melastomataceae and Rubiaceae (Homeier et al., 2008). The highest site at 3,000 m a.s.l. (S04 °06′711″, W79 °10′58″) is near the upper Cajanuma mountain at the northwest gate of Podocarpus National Park. The forest has been classified as an evergreen elfin forest dominated by trees/shrubs of Aquifoliaceae, Bromeliaceae, Chloranthaceae, Clusiaceae, Ericaceae and Melastomataceae (Homeier et al., 2008). The climate is semi-humid with an average annual temperature of 14.9, 12.3 and 8.9 °C and annual precipitation of approximately 2,200, 3,500 and 4,500 mm at 1,000, 2,000 and 3,000 m a.s.l., respectively (Bendix et al., 2006; Homeier et al., 2010). Soil types of the study sites are alumic Acrisol (1,000 m), Gley Cambisol (2,000 m) and Podzol (3,000 m) (Soethe, Lehmann & Engels, 2006; Moser, Hertel & Leuschner, 2007). The thickness of the organic soil layers increases with altitude from 4.8 cm at 1,000 m to 30.5 cm at 2,000 m to 43.5 cm at 3,000 m (Leuschner et al., 2007; Graefe, Hertel & Leuschner, 2008). In parallel, fine root biomass increases from 2.7 to 6.2 to 10.8 t ha^−1^ at the respective sites (Soethe, Lehmann & Engels, 2006).

### Experimental design

Nylon litterbags (17 × 17 cm) with mesh sizes of 45 µm, 1 mm and 4 mm were filled with 10 g of leaf or root litter. The mesh sizes we used are typically used to evaluate the role of meso- and macrofauna in decomposition processes. The 45 µm mesh prevents access by even the smallest mites and springtails, the 1 mm mesh allows access by virtually all mesofauna species and the 4 mm mesh allows access by virtually all macrofauna species (Bradford et al., 2002; Kampichler & Bruckner, 2009; Bokhorst & Wardle, 2013). Leaf litter consisted of a mixture of freshly fallen leaves of three locally abundant tree species of each study site: *Pouteria* sp., *Cecropia andina* and *Mollinedia* sp. at 1,000 m, *Graffenrieda emarginata*, *Clusia* sp. and *Cavendishia zamorensis* at 2,000 m, *Clusia* sp. *Graffenrieda emarginata* and *Hedyosmum* sp. at 3,000 m. Root litter were collected by hand from the upper 20–30 cm of the soil/organic layer of respective sites and consisted of a mixture of three size classes: Small (<1 mm diameter), medium (1–2 mm diameter) and large (>3 mm diameter). The amount of individual leaf species and root size classes placed in the litterbags was chosen to resemble their amount in the litter layer and soil, respectively (see SI 1). This was done to mimic close to natural conditions in the litterbags for the colonization by the local soil animal community. The collected leaves and roots were gently rinsed with tap water to clear them from adhering soil and dried at 60 °C.

Litterbags were placed in the field in October 2008 (end of the rainy season). Bags containing leaf litter were randomly placed on top of the litter layer and fixed with nails, while those containing root litter were placed approximately 5 cm below the litter layer. Three blocks were established at each of the three altitudes with a minimum distance between blocks of 20 m. Two replicates of each treatment were placed in each block, with one replicate retrieved after 6 months and the other after 12 months.

After retrieval, the litter material in each litterbag was divided into two parts of equal mass. The first half was analyzed for dry mass, microbial biomass, basal respiration, and C and N concentrations. From the second half, Oribatida and Collembola were extracted using a modified high-gradient heat extractor (Macfadyen, 1961; Kempson, Lloyd & Ghelardi, 1963) and counted. Adult Oribatida were identified to species level or sorted into morphospecies (Balogh & Balogh, 1990; Balogh & Balogh, 2002), following the nomenclature of Subías (2018). All identified species are recorded in the Ecotaxonomy database (Potapov, Sandmann & Scheu, 2019).

### Analytical procedures

Mass loss (M_loss_) for both leaves and roots were calculated as *M*_loss_ = ((*m*_0_ − *m*_1_/*m*_0_)) × 100, where m_0_ is the dry weight of the initial litter placed in the litterbags and m_1_ the dry weight of litter at harvest. To measure carbon (C) and nitrogen (N) concentrations, dried (60 °C, 72 h) leaves and roots were milled to powder (<1 mm) and analyzed using a CN elemental analyzer (Vario EL III, Elementar, Hanau, Germany).

Microbial basal respiration (BR) and microbial biomass (C_mic_) were measured using a computer-controlled O_2_ micro-compensation apparatus (Scheu, 1992). BR (µl O_2_ g^−1^ dry weight h^−1^) was determined as mean O_2_ consumption rates 10 to 20 h after attachment of the samples to the respirometer. C_mic_ was calculated from the maximum initial respiratory response (MIRR; µl O_2_ g^−1^ h^−1^) measured after glucose saturation following the substrate-induced respiration method (SIR) of Anderson & Domsch (1978). MIRR was calculated as the average of the lowest three readings within the first 10 h and C_mic_ was calculated as C_mic_ = 38 × MIRR (mg g^−1^ dry weight) (Beck et al., 1997; Joergensen & Scheu, 1999).

### Statistical analyses

Analyses were performed using R version 3.6.0 (R Core Team, 2019). Linear mixed-effects models were used to analyze changes in M_loss_, C_mic_ , BR, the abundance of Oribatida and Collembola, and Oribatida species richness data separately for leaf and root litter (hypotheses 1 and 2). Sampling date (6 and 12 months), mesh size (45 µm, 1 mm and 4 mm), altitude (1,000, 2,000 and 3,000 m a.s.l.) and all possible interactions were fitted as fixed factors, and block was fitted as a random factor with random intercept (R package “nlme”; Pinheiro et al., 2020). Data were log-transformed if necessary. Model residuals were checked for normality and homoscedasticity using Shapiro–Wilk test and Bartlett’s test (R package “stats” R Core Team, 2019). Differences between means were inspected using Tukey’s honestly significant difference test (R package “emmeans”; Lenth, 2020). Pearson correlation coefficients (package “stats”) were calculated to investigate relationships between M_loss_, C_mic_ , BR, C-to-N ratio, and the abundance of Collembola and Oribatida, and Oribatida diversity separately for leaf and root litter (hypothesis 3).

Canonical correspondence analysis (CCA) performed in CANOCO 5.02 (Ter Braak & Smilauer, 2012) was used to explore the relationship between Oribatida community composition and litter characteristics (M_loss_, C-to-N ratio) as well as microbial indicators (BR, C_mic_ ) (hypothesis 4). Only species with more than three individuals in the samples were included. Monte Carlo randomization tests using 999 simulations were used to determine the significance of the axes. Sampling date (6 and 12 months), mesh sizes (45 µm, 1 mm and 4 mm) and altitude (1,000, 2,000 and 3,000 m a.s.l.) were coded as supplementary variables not affecting the ordination. Since the global test with all litter and microbial indicators was significant, we used forward selection to identify the most important variables structuring Oribatida communities. This was done to reduce the number of explanatory variables entering the analysis while keeping the variation explained caused by them at a maximum. The forward selection procedure was stopped if a variable reached a level of significance > 0.05 and *R*^2^ > 0.90 (Legendre & Legendre, 1998; Blanchet, Legendre & Borcard, 2008).

## Results

### Decomposition of leaves and roots

**Figure 1:**
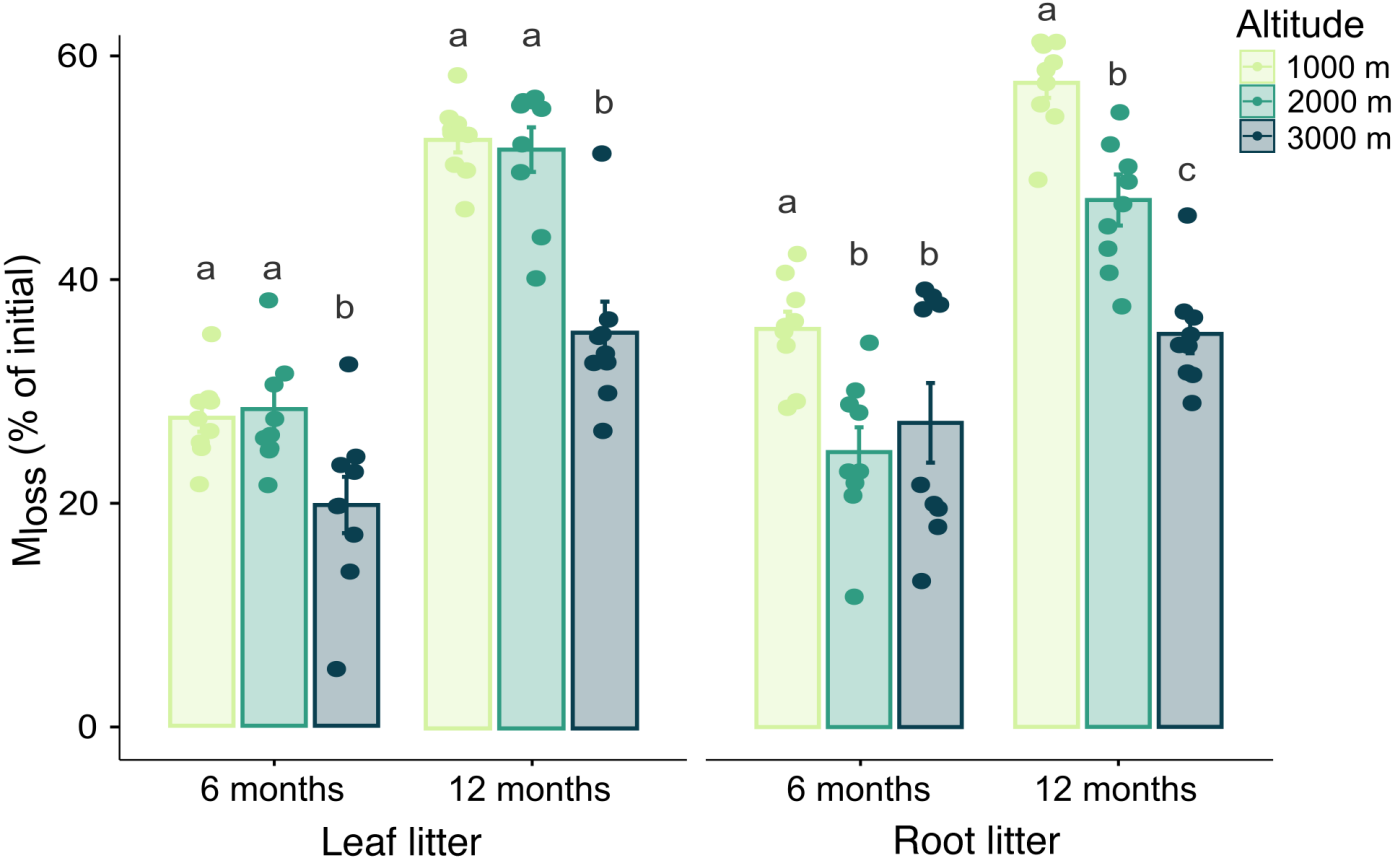
Effect of altitude on mass loss (M_loss_) after 6 and 12 months. Variations in M_loss_ of leaf and root litter exposed in litterbags at three different altitudes (1,000, 2,000 and 3,000 m a.s.l.) for 6 and 12 months. Values are means ± SE. For each litter type, bars marked with different letters within each time of exposure differ significantly (Tukey’s HSD tests, *p* < 0.05).

Generally, M_loss_ significantly increased during the time of exposure in both leaf and root litter. Leaf litter M_loss_ was not significantly affected by any interaction between the three factors studied, but it was higher at 1,000 and 2,000 m compared to 3,000 m at both sampling dates (Table 1, SI 2). By contrast, in root litter the interactions between altitude and date, as well as between altitude and mesh size significantly affected M_loss_. After 12 months it decreased in a linear way with increasing altitude (Fig. 1, Table 1). Further, at 3,000 m root litter M_loss_ in litterbags of 4 mm mesh size was higher than in litterbags of 45 µm and 1 mm mesh size (Table 1, SI 2). M_loss_ positively correlated with C_mic_ , BR and negatively with litter C-to-N ratios in both leaf and root litter. In root litter, M_loss_ also positively correlated with Collembola and Oribatida abundance and Oribatida richness (Table 2).

The C-to-N ratio of both leaf and root litter significantly increased with altitude from 1,000 m to similar values at 2,000 m and 3,000 m (Table 1, SI 2). In leaf litter the C-to-N ratio decreased with time, whereas the C-to-N ratio in root litter did not change significantly with time. In leaf litter mesh size did not affect the C-to-N ratio, whereas the C-to-N ratio in root litter was lower in litterbags with 45 µm mesh size. C-to-N ratios negatively correlated with M_loss_ and the abundance of Collembola and Oribatida, and Oribatida richness in leaf litter. In root litter, C-to-N ratios negatively correlated with M_loss_, C_mic_ , BR and the abundance of Collembola and Oribatida, and Oribatida richness (Table 2).

**Table 1:** Effects of time, mesh size and altitude on mass loss (M_loss_), litter C-to-N ratio, microbial biomass (C_mic_) and basal respiration (BR) in leaf and root litter. *F*- and *P*-values of linear mixed-effects models on the effect of time of exposure (6 and 12 months), mesh size (45 µm, 1 mm, 4 mm) and altitude (1,000, 2,000 and 3,000 m a.s.l.) on M_loss_, litter C-to-N ratio, C_mic_ and BR in leaf and root litter.

	**M_loss_**		**C-to-N**		**C_mic_**		**BR**	
	*F*-value	*p*-value	*F*-value	*p*-value	*F*-value	*p*-value	*F*-value	*p*-value
**Leaf litter**								
Time	**152.01**	**<0.001**	**21.41**	**<0.001**	**21.82**	**<0.001**	**39.37**	**<0.001**
Mesh size	0.069	0.527	1.05	0.362	**20.21**	**<0.001**	**6.98**	**0.003**
Altitude	**24.33**	**<0.001**	**233.30**	**<0.001**	**16.74**	**<0.001**	**12.82**	**<0.001**
Time × mesh size	1.42	0.255	0.02	0.980	0.30	0.741	**5.47**	**0.009**
Time × altitude	2.84	0.072	2.74	0.078	**8.17**	**0.001**	2.46	0.104
Mesh size × altitude	0.55	0.697	0.29	0.882	1.58	0.201	0.76	0.561
Time × mesh size × Altitude	1.13	0.360	0.24	0.911	**3.67**	**0.013**	**8.16**	**<0.001**
**Root litter**								
Time	**110.09**	**<0.001**	0.06	0.806	**167.86**	**<0.001**	**226.11**	**<0.001**
Mesh size	0.79	0.461	**4.46**	**0.019**	1.62	0.213	0.96	0.391
Altitude	**30.19**	**<0.001**	**106.07**	**<0.001**	**128.27**	**<0.001**	**32.67**	**<0.001**
Time × mesh size	0.21	0.808	0.97	0.388	0.90	0.416	2.17	0.129
Time × altitude	**8.01**	**0.001**	0.99	0.382	**18.33**	**<0.001**	1.14	0.332
Mesh size × altitude	**3.26**	**0.023**	1.61	0.193	**6.81**	**<0.001**	**6.19**	**<0.001**
Time × mesh size × Altitude	1.67	0.179	2.42	0.067	**5.47**	**0.001**	1.16	0.343

**Notes.**

Significant effects are given in bold, *p* < 0.05.

### Microorganisms

Generally, C_mic_ and BR significantly increased with time of exposure and varied with altitude in both litter types (Table 1, SI 3). In leaf litter, C_mic_ was higher at 1,000 and 2,000 m compared to 3,000 m, whereas in root litter, both C_mic_ and BR were higher at 1,000 m compared to 2,000 and 3,000 m.

**Figure 2:**
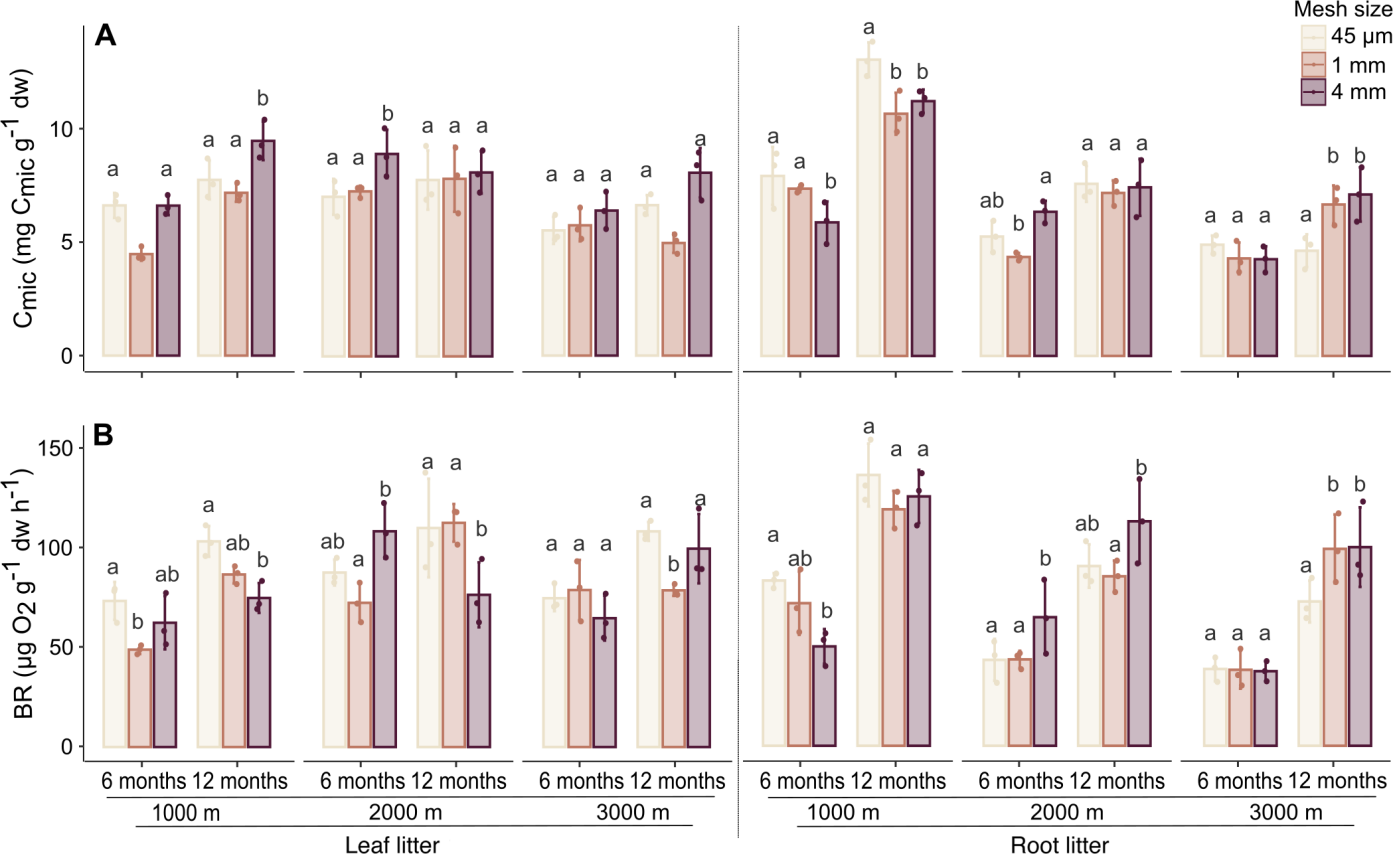
Effect of mesh size and altitude on C_mic_ and BR after 6 and 12 months. Variation in (A) microbial biomass (C_mic_) and (B) basal respiration (BR) in leaf litter (left panel) and root litter (right panel) at three altitudes (1,000, 2,000 and 3,000 m a.s.l.) after 6 and 12 months of incubation. Values are means ± SE. For each litter type, altitude and sampling date, bars marked with different letters differ significantly among mesh sizes (Tukey’sHSD tests, *p* < 0.05).

In leaf litter, variations in C_mic_ and BR with altitude depended on time, but the effect of altitude also varied with mesh size (significant three factor interaction; Fig. 2, Table 1, SI 3). Similar to leaf litter, in root litter variations in C_mic_ with altitude depended on time and mesh size while variations in BR in root litter with altitude depended on mesh size (Fig. 2, Table 1, SI 3). In both leaf and root litter, C_mic_ and BR positively correlated with M_loss_ (Table 2). In root litter, C_mic_ and BR also positively correlated with Collembola and Oribatida abundances, and Oribatida richness, but negatively with litter C-to-N ratios.

### Abundance of Collembola and Oribatida

Contrasting C_mic_ and BR, time of exposure as main effect neither affected the abundance of Collembola nor that of Oribatida (Table 3). Rather, the abundance of Collembola and Oribatida in both litter types varied strongly with altitude and mesh size.

Generally, in both litter types, the abundance of Collembola decreased strongly with increasing altitude (Table 3, SI 5). In leaf litter the decrease in Collembola abundance with altitude also varied with sampling date. In root litter, the variation in the abundance of Collembola with altitude varied significantly with mesh size (Table 3, SI 5 and SI 6).

Generally, contrasting the pattern in Collembola, the abundance of Oribatida in leaf litter was similar at 1,000 and 2,000 m and significantly lower at 3,000 m, whereas in root litter the abundance at 1,000 m strongly exceeded that at 2,000 and 3,000 m (Table 3, SI 5). In contrast to Collembola, in both litter types, the abundance of Oribatida generally varied with mesh size. However, in root litter the effect varied with altitude (Table 3, SI 5 and SI 6).

In both leaf and root litter the abundance of Collembola and Oribatida correlated negatively with litter C-to-N ratios and positively with the Oribatid richness (Table 2). However, in root litter the abundance of Collembola and Oribatida also correlated positively with M_loss_, C_mic_ and BR.

**Table 2:** Pearson correlation coefficients between mass loss (M_loss_), microbial biomass (C_mic_), basal respiration (BR), litter C-to-N ratio, the abundance of Collembola, and the abundance and richness of Oribatida in leaf and root litter.

	**M_loss_**	**C_mic_**	**BR**	**Collembola abundance**	**Oribatida abundance**	**Oribatida richness**
**Leaf litter**						
C_mic_	**0.45** ^***^	1	–	–	–	–
BR	**0.32** ^*^	**0.55** ^***^	1	–	–	–
Collembola abundance	0.12	0.15	−0.24	1	–	–
Oribatida abundance	0.11	0.11	−0.16	**0.50** ^**^	1	–
Oribatida richness	0.19	0.14	−0.20	**0.54** ^***^	**0.92** ^***^	1
C-to-N	**−0.44** ^***^	−0.11	0.18	**−0.60** ^***^	**−0.31** ^*^	**−0.45** ^***^
**Root litter**						
C_mic_	**0.75** ^***^	1	–	–	–	–
BR	**0.74** ^***^	**0.86** ^***^	1	–	–	–
Collembola abundance	**0.35** ^**^	**0.46** ^***^	**0.35** ^**^	1	–	–
Oribatida abundance	**0.33** ^**^	**0.44** ^***^	**0.33** ^**^	**0.67** ^***^	1	–
Oribatida richness	**0.36** ^***^	**0.45** ^***^	**0.35** ^**^	**0.68** ^***^	**0.98** ^***^	1
C-to-N	**−0.47** ^***^	**−0.64** ^***^	**−0.41** ^***^	**−0.43** ^***^	**−0.49** ^***^	**−0.52** ^***^

**Notes.**

Significant correlations are given in bold (* *P* < 0.05; ** *P* < 0.01; *** *P* < 0.001).

**Table 3:** Effects of time, mesh size and altitude on the abundance of Collembola, and the abundance and richness of Oribatida in leaf and root litter. *F*- and *P*-values of linear mixed-effects models on the effect of time of exposure (6 and 12 months), mesh size (45 µm, 1 mm, 4 mm) and altitude (1,000, 2,000 and 3,000 m a.s.l.) on the abundance of Collembola, and the abundance and richness of Oribatida in leaf and root litter.

	**Collembola**		**Oribatida**			
	**Abundance**		**Abundance**		**Richness**	
	*F*-value	*p*-value	*F*-value	*p*-value	*F*-value	*p*-value
**Leaf litter**						
Time	0.09	0.756	0.07	0.783	0.04	0.841
Mesh size	**5.77**	**0.007**	**29.41**	**<0.001**	**32.13**	**<0.001**
Altitude	**26.22**	**<0.001**	**11.80**	**<0.001**	**21.21**	**<0.001**
Time × mesh size	0.15	0.857	2.64	0.085	**4.63**	**0.016**
Time × altitude	**3.25**	**0.051**	0.07	0.933	0.48	0.61
Mesh size × altitude	0.34	0.849	0.519	0.722	0.79	0.541
Time × mesh size × Altitude	2.14	0.097	2.41	0.068	**2.82**	**0.039**
**Root litter**						
Time	0.01	0.895	0.25	0.618	0.01	0.921
Mesh size	2.61	0.088	**33.47**	**<0.001**	**37.86**	**<0.001**
Altitude	**53.07**	**<0.001**	**64.88**	**<0.001**	**70.23**	**<0.001**
Time × mesh size	0.14	0.870	0.34	0.71	0.75	0.481
Time × altitude	1.59	0.217	1.78	0.18	2.25	0.121
Mesh size × altitude	**3.81**	**0.011**	**6.01**	**<0.001**	**3.57**	**0.015**
Time × mesh size × Altitude	1.92	0.129	0.73	0.57	0.77	0.553

**Notes.**

Significant effects are given in bold, *p* < 0.05.

### Oribatida species richness

In both leaf and root litter the average Oribatida species richness per litterbag was significantly affected by mesh size and altitude (Table 3, SI 5). In leaf litter, Oribatida species richness was generally affected by the interaction between time, altitude and mesh size; whereas in root litter Oribatida species richness only varied with mesh size and altitude (Table 3, SI 5). In both leaf and root litter Oribatida species richness correlated negatively with litter C-to-N ratios and positively with the abundance of Collembola and Oribatida (Table 2). However, in root litter Oribatida richness correlated positively with M_loss_, C_mic_ and BR.

### Community structure of Oribatida

**Figure 3:**
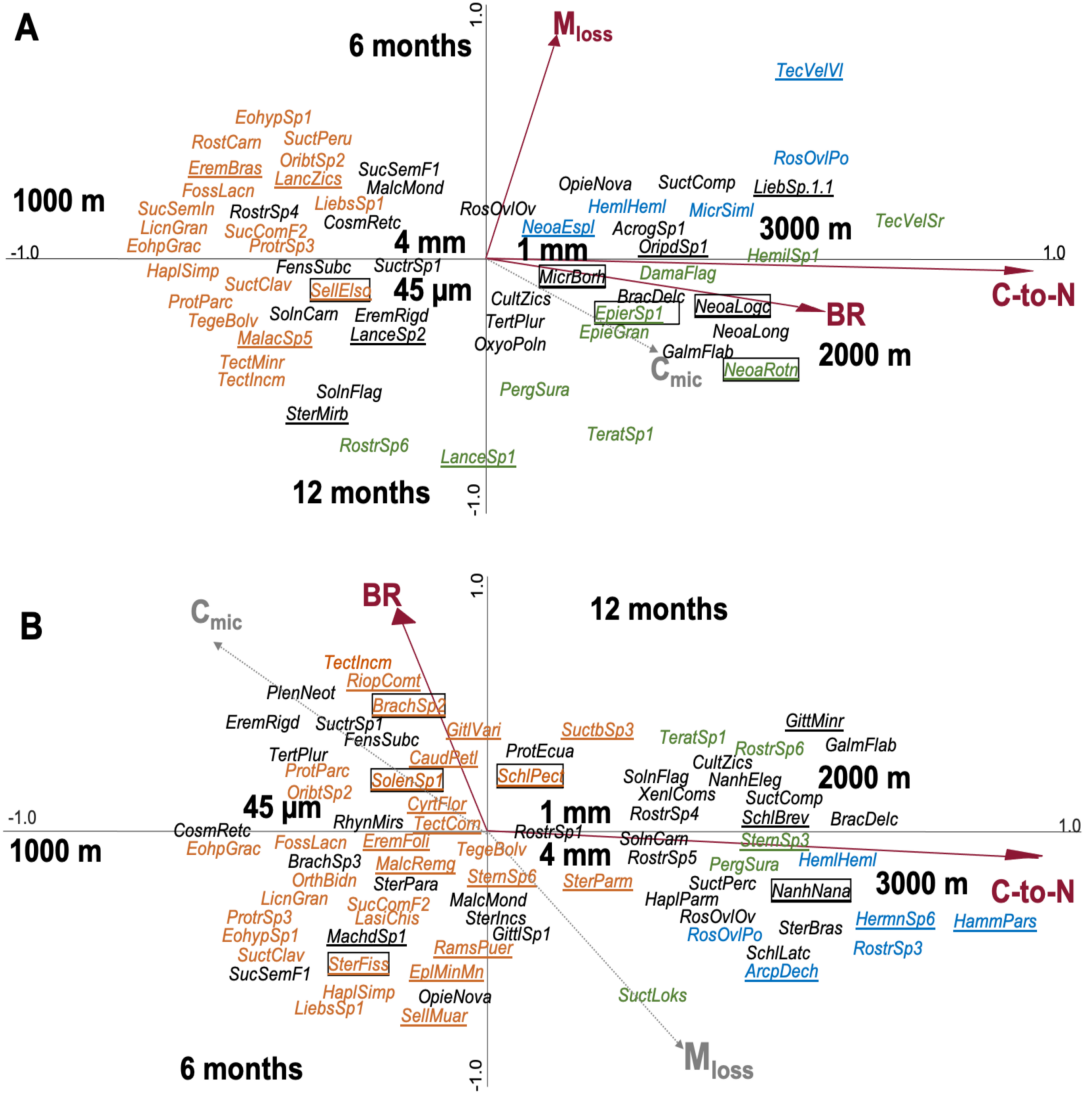
Canonical correspondence analysis (CCA) of Oribatida species in litterbags with (A) leaf and (B) root litter and their relationship with environmental variables (forward selection). Arrows in red represent significant environmental variables. Species present only at one of the three altitudes are marked in color (orange = 1,000 m, green = 2,000 m, blue = 3,000 m), others are given in black. Species present in only one litter type are underlined and of them the most abundant species (>10 individual across the samples) are framed; for full species names see SI 7.

In total, 176 species of Oribatida were identified (see SI 7 for full list of species). CCA of Oribatida species in leaf litter with litter C-to-N ratio, M_loss_, BR and C_mic_ included as environmental variables explained 12.7% of the variation of Oribatida community composition (Fig. 3A); litter C-to-N ratio accounted for 4.4% (pseudo-F = 1.9, *P* = 0.002), M_loss_ for 4.3% (pseudo-F = 1.8, *P* = 0.002) and BR for 3.9% (pseudo-F = 1.6, *P* = 0.007). The community composition of Oribatida at 2,000 and 3,000 m correlated positively with increasing litter C-to-N ratio and BR; M_loss_ correlated positively with the first sampling date and was associated with lower species abundance.

CCA of Oribatida species in root litter with litter C-to-N ratio, BR, M_loss_ and C_mic_ included as environmental variables explained 10.2% of the variation of Oribatida community composition (Fig. 3B); litter C-to-N ratio accounted for 7.0% of the variation (pseudo-F = 3.7, *P* < 0.001) and BR for 3.2% (pseudo-F = 1.6, *P* = 0.004). As in leaf litter, Oribatida species at 2,000 and 3,000 m correlated positively with increasing litter C-to-N ratio, but BR correlated positively with the sampling date after 12 months. In both the CCA of leaf and root litter, the 1,000 m site was separated from the 2,000 and 3,000 m sites along the first axis and the centroids of mesh size were close to the center of the ordination. As note of caution, however, most Oribatida species were rare and only occurred at certain altitudes and at specific sampling dates; for the abundance of species at each of the study sites see SI 7.

## Discussion

### Variations in leaf and root litter decomposition with altitude

In contrast to our first hypothesis, M_loss_ of both leaf and root litter showed different patterns of decomposition along the altitudinal gradient. For leaf litter, M_loss_ did not follow the expected linear decrease with altitude, rather, decomposition rates at 1,000 and 2,000 m were similar after the 12 months of exposure. This contrasts previous studies at our study sites (Illig et al., 2008; Marian et al., 2017; Marian et al., 2019) and indicates that leaf litter decomposition cannot be explained only by the linear decrease in temperature along the altitudinal gradient studied. Potentially, the decline in leaf litter decomposition with temperature was compensated by higher precipitation at 2,000 m compared to 1,000 m (see Methods). High rainfall facilitates decomposition especially at early stages by increasing leaching of soluble compounds (Cusack et al., 2009). However, although climate is considered the primary driver of litter decomposition at large scales (Coûteaux, Bottner & Berg, 1995; Aerts, 1997), the role of climatic factors might be overridden by the variability of litter traits at local scales (Scowcroft, Turner & Vitousek, 2000; Richardson, Richardson & Soto-Adames, 2005; Fujii et al., 2017). In our study, litter characteristics such as C-to-N ratio differed strongly between the leaf litter materials exposed at the three altitudes. However, as indicated by the C-to-N ratio, leaf litter materials from the 1,000 m site were of considerably higher quality than those from the 2,000 m site (as well as the 3,000 m site), suggesting that the high decomposition rates of leaf litter at 2,000 m also cannot be explained by litter quality. Nonetheless, caution is recommended in the interpretation of this result, as other litter chemical compounds were not measured and might have influenced litter decomposition rates. Further, leaf litter M_loss_ was potentially modified by physicochemical interactions among the three leaf litter species placed in the litterbags and biotic factors such as microbial community composition. The fact that C_mic_ and BR were higher in leaf litter at 2,000 m than at 1,000 m after 6 months of exposure supports this conclusion and suggests that the leaf litter mixtures favoured the activity and abundance of microbial communities early after exposure. The generally high values of C_mic_ and BR in leaf litter at 2,000 and 3,000 m, despite the very high litter C-to-N ratio, also suggest that microbial communities at these sites are well adapted to decompose litter of low quality (Gholz et al., 2000; Strickland et al., 2009; Milcu & Manning, 2011; Marian et al., 2017).

In contrast to leaf litter, root litter showed the expected linear decrease in litter decomposition with increasing altitude after 12 months. Less favorable abiotic conditions, such as those at higher altitude, might affect root litter decomposition by reducing the quality of the litter material and thereby nutrient availability as reported for tropical rainforests (Vitousek et al., 1994; Tanner, Vltousek & Cuevas, 1998; Kitayama et al., 2004). This is supported by the high C-to-N ratios and low C_mic_ and BR, as well as the abundance of decomposer microarthropods at 2,000 and 3,000 m (compared to 1,000 m). The contrasting results between leaf and root litter decomposition rates suggest that in the studied tropical montane rainforests differences in litter chemical composition and their association with nutrient limitations among the altitudinal sites are more critical factors for the decomposition of root litter than for leaf litter. Nonetheless, a complete litter chemical composition profile is needed to support this conclusion. Moreover, leaf litter might be more susceptible than roots to effects of climatic variations as leaf litter is located on top of the soil and thereby exposed to more variable microclimatic conditions than root litter in soil (Ostertag & Hobbie, 1999; Silver & Miya, 2001). However, as root litter generally decomposed slower than leaf litter at 2,000 and 3,000 m, more buffered conditions in soil do not implicate an override of the primacy of nutrient limitations as driving factor of litter decomposition. Nonetheless, at 1,000 m the more buffered climatic condition together with the proximity of the mineral soil layer might have favoured the faster decomposition rates of roots than leaf litter.

### Faunal contribution to leaf and root litter decomposition

The abundance of both Collembola and Oribatida was higher in 1 and 4 mm mesh bags irrespective of the plant litter type indicating that, as intended, 45 µm mesh size restricted the access to the litterbags by mesofauna. Thus, the different mesh sizes are a useful tool to evaluate the effects of decomposer animals on decomposition processes. However, restricting the access of mesofauna in 45 µm litterbags was more effective in Oribatida than in Collembola, indicating that the mesh size approach is limited for evaluating the role of mesofauna for decomposition processes and suggesting that it likely underestimates their effects on litter decomposition as discussed earlier (Bradford et al., 2002; Kampichler & Bruckner, 2009). Further, contrasting our second hypothesis, access by microarthropods to litterbags containing leaf litter did not vary the general decomposition rates, nor at any of the three altitudes. However, soil microarthropod access to root litter increased its mass loss at 3,000 m (4 mm mesh bags). Despite the widely assumed beneficial effects of soil microarthropods on litter decomposition, experimental evidence supporting this assumption is controversial; some studies indeed found positive effects on litter mass loss (Bradford et al., 2002; Carrillo et al., 2011; Bokhorst & Wardle, 2013), whereas others suggest their contribution to be minor or absent (Schinner, 1982; Joo, Yim & Nakane, 2003; Kampichler & Bruckner, 2009; Marian et al., 2019). Overall, our results for leaf litter support the latter and previous findings at our study sites also indicate that the decomposition of leaf litter is predominantly due to microorganisms, with the contribution of microarthropods being minor, in particular at early stages of litter decomposition (Illig et al., 2008; Marian et al., 2017; Marian et al., 2019). This finding is supported by lack of correlation of Collembola and Oribatida abundances with M_loss_, C_mic_ and BR in leaf litter. Both decomposer groups may play a more important role at more advanced stages of decomposition when microorganisms have colonized leaf litter making it more palatable for arthropod consumers (Bardgett, 2005; Coulis et al., 2009; Das & Joy, 2009). However, the increase in root litter M_loss_ at 3,000 m in 4 mm mesh bags suggests that under unfavourable environmental conditions, the decomposition of low-quality litter is stimulated by soil arthropods. Several processes may have accelerated root litter decomposition including stimulation of microbial growth *via* nutrient mobilization, litter fragmentation and dispersal of microbial propagules (Verhoef & Brussaard, 1990; Ruess & Lussenhop, 2005; Scheu, Ruess & Bonkowski, 2005). The fact that at 3,000 m C_mic_ and BR in roots increased in 1 and 4 mm mesh bags supports these findings and reinforces that the contribution of microarthropods to decomposition of recalcitrant substrates is more pronounced than in readily decomposable materials (Joo, Yim & Nakane, 2006; Milcu & Manning, 2011; Gergócs & Hufnagel, 2016).

Notably, C_mic_ and BR varied with mesh size in both leaf and root litter with the effect in root but not in leaf litter varying with altitude. Generally, C_mic_ was higher in 1 and 4 mm mesh bags, while BR was higher in 45 µm mesh bags. Overall, this supports our expectation that decomposer microarthropods stimulate microorganisms, thereby enhancing their biomass. Potentially, grazing on microorganisms results in increased nutrient mobilization favouring microbial growth (Seastedt, 1984; Hättenschwiler, Tiunov & Scheu, 2005). Further, grazing may foster changes in microbial community composition resulting in more effective use of resources by microorganisms and this may explain the reduced BR in litterbags with coarse mesh size (Dilly & Munch, 1996; Chapman et al., 2013). Indeed, it has been stressed earlier that the structure of microbial communities is an important determinant of litter decomposition rates particularly in forest ecosystems (Strickland et al., 2009).

Additionally, despite the general lack of effect of mesh size on root litter mass loss, the abundances of both Collembola and Oribatida, and Oribatida species richness positively correlated with M_loss_, C_mic_ and BR. These results support our third hypothesis and suggest that litter accumulation in nutrient-poor sites can provide more habitat space for soil decomposer microarthropods. Notably, the increase in animal abundance and diversity may enhance the faunal contribution to litter decomposition (Perez et al., 2013). Moreover, the close vicinity of roots to the mineral soil layer might have favored nutrient availability and thereby improved food resources for decomposer microarthropods (Marian et al., 2019). Further, at our study sites, the role of root exudates and mycorrhizal fungi are increasingly recognized as drivers of litter decomposition, mineralization processes and determinants of soil food webs (Marian et al., 2019; Sánchez-Galindo et al., 2019). Therefore, at 3,000 m, where the concentration of root biomass is at a maximum (Röderstein, Hertel & Leuschner, 2005; Soethe, Lehmann & Engels, 2007), soil arthropods may benefit more from root-derived resources than at 1,000 and 2,000 m either by grazing on microorganisms or directly by feeding on roots.

Interestingly, the abundance of both decomposer microarthropods in leaf and root litter did not vary significantly with sampling date. Potentially, the nutritional value of the litter material for these detritivore microarthropods changed little during the exposure time. Substrate quality may also be related to the generally low effects of soil decomposer microarthropods on leaf and root litter decomposition. Litter of different quality may attract different faunal communities and this likely contributes to the varying effects of decomposer animals on decomposition processes (Fujii & Takeda, 2012; Fujii et al., 2017).

### Oribatida species richness and community structure in leaf and root litter

Similar to Oribatida abundance, the higher Oribatida species richness in litterbags of 1 and 4 mm mesh size in both litter types might be attributed to restricted access of microarthropods to the 45 µm mesh litterbags. Oribatida species richness mostly varied with altitude in both leaf and root litter. The significant decrease in Oribatida species richness with increasing altitude in leaf litter supports results of previous studies at our study sites in that species richness of Oribatida in leaf litter is driven predominantly by factors linked to altitude such as temperature, precipitation, moisture and soil pH (Illig et al., 2008; Marian et al., 2018). By contrast, in root litter, the high number of Oribatida species at 1,000 m and the similarly low numbers at 2,000 and 3,000 m suggest that apart from abiotic conditions changing with altitude other factors modify Oribatida species richness. The fact that C-to-N ratios of root litter were similar at 2,000 and 3,000 m support this conclusion and suggests that root litter quality may be a regulator of Oribatida richness; the strong correlation of Oribatida species richness with litter C-to-N ratio supports this conclusion and suggests that increasing amounts of low-quality root litter material with altitude detrimentally affect Oribatida richness at 2,000 and 3,000 m (Röderstein, Hertel & Leuschner, 2005; Maraun et al., 2013). Indeed, Illig et al. (2010) also concluded that Oribatida species diversity at the studied montane rainforests is related to litter quality as important driving factor.

Similar to Oribatida species richness, Oribatida community assemblages varied mostly with altitude in both leaf and root litter. Most of the 176 species identified were registered on the 1,000 m site, only few species were only present at 2,000 and 3,000 m, presumably reflecting less favorable climatic conditions and poor resource quality at the highest altitudes (Marian et al., 2018). Interestingly, in leaf litter certain Oribatida species including *Sternoppia mirbilis*, *Lanceoppia* sp.1 and *Rostrozetes* sp.6 preferentially colonized litter after 12 months of exposure with the latter two species exclusively recorded at 2,000 m. This supports our fourth hypothesis indicating that certain Oribatida species rely on food resources associated with changes in leaf litter physicochemistry during decomposition particularly at higher elevations, suggesting that Oribatida species diversity at least in part is due to resource partitioning (Marian et al., 2018). This is supported by our finding that litter C-to-N ratio works as important factor structuring Oribatida communities in both leaf and root litter. Contrasting leaf litter, increase in root litter decomposition did not affect Oribatida community structure, despite changes in C_mic_ and BR with time were more pronounced in root than in leaf litter. This suggests that in contrast to our expectations, Oribatida community structure in root litter is not principally linked with decomposition or the gross characteristics of microbial communities. However, potential effects of root litter decomposition on the general quality of the organic litter material may function as key factor structuring Oribatida communities as indicated by their relationship with C-to-N ratios. Additionally, as mentioned before, root exudates may also modify the general characteristics of the organic matter and become an important carbon source fuelling Oribatida communities (Pollierer et al., 2007; Dennis, Miller & Hirsch, 2010; Zieger et al., 2017). Overall, our results indicate that the environmental factors considered explained only little of the variation in Oribatida community composition in both leaf and root litter. Still, both leaf and root litter characteristics, such as C-to-N ratio, as well as C_mic_ , significantly correlated with Oribatida community composition and this is consistent with earlier findings (Fujii & Takeda, 2017; Marian et al., 2018).

## Conclusions

The results of our study suggest that the decomposition of both leaf and root litter in montane rainforests is mainly due to microorganisms when compared to the role of microarthropods. However, soil microarthropods may contribute to the decomposition of low-quality litter such as root litter, particularly at very high altitude. Generally, the contrasting patterns of microbial biomass and the abundance of decomposer microarthropods and Oribatida species richness with the time of exposure highlight the minor importance of microorganism abundance as a food resource and structuring force of decomposer microarthropod communities. Rather, the results point to the dominance of litter characteristics, such as litter C-to-N ratio, as key factor structuring Oribatida communities in both leaf and root litter. Additionally, our findings highlight the main role of climatic factors driving root litter decomposition across altitudinal gradients, but their effects on leaf litter decomposition might be overridden at the local scale by litter traits and biotic factors. Further, at local scales differences in litter characteristics and the nutritional requirements of decomposer communities may modulate both leaf and root litter decomposition, and nutrient cycling in tropical montane rainforest ecosystem. Importantly, however, our 12 months study may not have been long enough to uncover the total contribution of soil microarthropods to litter decomposition. Due to general low quality of the litter material in this tropical rainforest, the functional role of soil microarthropods might become more significant in the long-term. Nonetheless, the study contributed to a better understanding of the role of decomposer microarthropods for litter decomposition in tropical rainforest ecosystems.

##  Supplemental Information

10.7717/peerj.14264/supp-1 Data S1 Raw data used for statistical analysis

10.7717/peerj.14264/supp-2 Supplemental Information 2 Supplemental information

## Additional Information and Declarations

### Field Study Permissions

The following information was supplied relating to field study approvals (*i.e.*, approving body and any reference numbers): The Center Naturaleza y Cultura Internacional (NCI) allowed us to work in the San Francisco reserve with Research Permit No. 028-IC-FAU/FLO-DPZCH-MA We included this information in the acknowledgments section.

### Data Availability

The following information was supplied regarding data availability: The raw data is available in the Supplemental Files.
