# Supplementary material for: Differences in leaf and root litter decomposition in tropical montane rainforests are mediated by soil microorganisms not by decomposer microarthropods
Source: PeerJ. 2022 Nov 3;10:e14264. doi: 10.7717/peerj.14264 (PMC9637353; doi:10.7717/peerj.14264)
Supplement: Supplemental Information 2 [file peerj-10-14264-s002.docx]

**Supplementary Information (SI)**

**SI 1.** Leaf and root litter mixtures per litterbags at the three altitudes (1000, 2000 and 3000 m).

| **Site** | **Litter type** | **Species** | **Mass (g)** | **Total (g)** |
| --- | --- | --- | --- | --- |
| 1000 m | Leaves | *Pouteria* sp. | 5.0 | 10 |
|  |  | *Cecropia* sp. | 3.0 |  |
|  |  | *Mollinedia* sp. | 2.0 |  |
|  | Roots | Small | 2.7 | 10 |
|  |  | Medium | 4.8 |  |
|  |  | Large | 2.5 |  |
| 2000 m | Leaves | *Graffenrieda emarginata* | 5.0 | 10 |
|  |  | *Clusia* sp. | 4.0 |  |
|  |  | *Cavendishia zamorensis* | 1.0 |  |
|  | Roots | Small | 4.4 | 10 |
|  |  | Medium | 2.1 |  |
|  |  | Large | 3.5 |  |
| 3000 m | Leaves | *Clusia* sp. | 5.0 | 10 |
|  |  | *Graffenrieda emarginata* | 4.0 |  |
|  |  | *Hedyosmum* sp. | 1.0 |  |
|  | Roots | Small | 3.4 | 10 |
|  |  | Medium | 2.5 |  |
|  |  | Large | 4.1 |  |

**SI 2.** Mass loss (M_loss_) and C-to-N ratio in leaf and root litter in litterbags of 45 µm, 1 mm and 4 mm mesh exposed at 1000, 2000 and 3000 m for 6 and 12 months. Values are means ± SD.

|  | | | | **Leaf litter** | | **Root litter** | |
| --- | --- | --- | --- | --- | --- | --- | --- |
|  |  |  |  | **M_loss_**  **[% of initial]** | **C-to-N** | **M_loss_**  **[% of initial]** | **C-to-N** |
| **Time** | | | | | | | |
| 6 months | | | | 25.6 ± 7.1 | 53.99 ± 22.27 | 29.1 ± 8.8 | 57.64 ± 14.35 |
| 12 months | | | | 46.6 ± 10.1 | 44.94 ± 17.50 | 46.6 ± 10.7 | 57.20 ± 15.49 |
| **Mesh size** | | | | | | | |
| 45 µm | | | | 36.2 ± 13.5 | 51.20 ± 20.35 | 37.6 ± 13.6 | 53.71 ± 13.09 |
| 1 mm | | | | 36.4 ± 15.2 | 49.04 ± 22.03 | 36.7 ± 12.9 | 59.02 ± 16.01 |
| 4 mm | | | | 35.7 ± 12.9 | 48.14 ± 19.67 | 39.2 ± 13.5 | 59.53 ± 15.26 |
| **Altitude** | | | | | | | |
| 1000 m | | | | 40.3 ± 13.1 | 23.74 ± 2.86 | 46.6 ± 12.1 | 39.54 ± 4.10 |
| 2000 m | | | | 40.3 ± 13.2 | 59.54 ± 11.70 | 35.8 ± 13.3 | 63.99 ± 9.68 |
| 3000 m | | | | 27.7 ± 11.1 | 65.10 ± 8.95 | 31.2 ± 9.1 | 68.99 ± 9.67 |
| **Time × mesh size** | | | | | | | |
| 6 months | | 45 µm | | 27.5 ± 9.2 | 55.83 ± 21.93 | 28.7 ± 9.1 | 55.30 ± 12.37 |
|  |  | 1 mm | | 24.2 ± 7.9 | 53.28 ± 24.67 | 27.4 ± 8.35 | 59.48 ± 17.35 |
|  |  | 4 mm | | 25.2 ± 3.4 | 52.86 ± 22.73 | 31.3 ± 9.65 | 58.14 ±14.33 |
| 12 months | | 45 µm | | 44.9 ± 11.4 | 46.56 ± 18.75 | 45.6 ± 11.5 | 52.12 ± 14.33 |
|  |  | 1 mm | | 48.6 ± 9.7 | 44.80 ± 19.57 | 45.9 ± 9.6 | 58.57 ± 15.58 |
|  |  | 4 mm | | 46.2 ± 9.7 | 43.44 ± 15.98 | 47.2 ± 12.2 | 60.92 ± 16.89 |
| **Time × altitude** | | | | | | | |
| 6 months | | 1000 m | | 28.0 ± 3.8 | 25.12 ± 2.57 | 35.6 ± 4.6 | 41.15 ± 3.34 |
|  |  | 2000 m | | 28.8 ± 6.3 | 67.52 ± 10.37 | 24.6 ± 6.6 | 62.32 ± 7.65 |
|  |  | 3000 m | | 20.1 ± 7.7 | 69.31 ± 9.72 | 27.2 ± 10.7 | 69.45 ± 10.60 |
| 12 months | | 1000 m | | 52.6 ± 3.4 | 22.34 ± 2.52 | 57.6 ± 4.0 | 37.94 ± 4.34 |
|  |  | 2000 m | | 51.7 ± 6.0 | 51.56 ± 6.37 | 47.1 ± 6.8 | 62.12 ± 6.52 |
|  |  | 3000 m | | 35.4 ± 8.3 | 60.91 ± 6.18 | 35.1 ± 5.2 | 68.54 ± 9.28 |
| **Mesh size × altitude** | | | | | | | |
| 45 µm | | 1000 m | | 42.3 ± 13.3 | 25.25 ± 3.19 | 48.2 ± 13.5 | 37.52 ± 3.92 |
|  |  | 2000 m | | 40.1 ± 13.3 | 61.37 ± 8.90 | 34.9 ± 12.4 | 62.74 ± 5.34 |
|  |  | 3000 m | | 26.2 ± 8.7 | 66.98 ± 9.41 | 29.7 ± 8.7 | 60.87 ± 8.04 |
| 1 mm | | 1000 m | | 40.9 ± 14.5 | 21.99 ± 2.99 | 44.8 ± 11.0 | 40.16 ± 4.27 |
|  |  | 2000 m | | 39.7 ± 13.3 | 59.59 ± 15.87 | 39.1 ± 11.7 | 63.77 ± 9.87 |
|  |  | 3000 m | | 28.7 ± 17.1 | 65.55 ± 9.09 | 26.2 ± 9.5 | 73.13 ± 7.88 |
| 4 mm | | 1000 m | | 37.7 ± 13.6 | 23.99 ± 1.50 | 46.8 ± 13.5 | 40.96 ± 3.99 |
|  |  | 2000 m | | 41.0 ± 15.4 | 57.65 ± 12.53 | 33.4 ± 16.9 | 64.64 ± 6.38 |
|  |  | 3000 m | | 28.3 ± 6.3 | 62.79 ± 9.51 | 37.6 ± 5.9 | 72.98 ± 8.53 |
| **Time × mesh size × Altitude** | | | | | | | |
| 6 months | 45 µm | | 1000 m | 30.6 ± 4.5 | 27.52 ± 3.009 | 36.5 ± 6.7 | 40.39 ± 1.99 |
|  |  |  | 2000 m | 30.5 ± 11.4 | 68.11 ± 3.10 | 24.1 ± 4.2 | 65.59 ± 6.50 |
|  |  |  | 3000 m | 21.4 ± 10.1 | 71.87 ± 9.48 | 25.4 ± 11.3 | 59.93 ± 6.44 |
|  | 1 mm | | 1000 m | 27.7 ± 1.9 | 23.46 ± 1.13 | 35.1 ± 0.9 | 42.30 ± 5.42 |
|  |  |  | 2000 m | 28.8 ± 2.9 | 68.15 ± 16.81 | 29.1 ± 5.8 | 57.74 ± 11.17 |
|  |  |  | 3000 m | 16.1 ± 9.6 | 68.25 ± 12.22 | 18.1 ± 4.5 | 78.38 ± 8.13 |
|  | 4 mm | | 1000 m | 25.69 ± 3.9 | 24.47 ± 1.47 | 35.1 ± 6.1 | 40.76 ± 2.87 |
|  |  |  | 2000 m | 27.11 ± 3.4 | 66.30 ± 11.62 | 20.5 ± 8.3 | 63.61 ± 4.13 |
|  |  |  | 3000 m | 22.74 ± 2.4 | 67.82 ± 10.76 | 38.1 ± 0.9 | 70.03 ± 9.25 |
| 12 months | 45 µm | | 1000 m | 54.01 ± 4.1 | 22.98 ± 0.69 | 59.9 ± 2.0 | 34.66 ± 3.13 |
|  |  |  | 2000 m | 49.79 ± 5.9 | 54.63 ± 7.24 | 45.8 ± 3.8 | 59.90 ± 2.15 |
|  |  |  | 3000 m | 31.05 ± 5.1 | 62.09 ± 7.40 | 34.0 ± 2.6 | 61.81 ± 10.84 |
|  | 1 mm | | 1000 m | 54.07 ± 0.5 | 20.53 ± 3.82 | 54.4 ± 5.0 | 38.02 ± 1.58 |
|  |  |  | 2000 m | 50.57 ± 8.9 | 51.04 ± 7.12 | 49.2 ± 2.7 | 69.80 ± 3.11 |
|  |  |  | 3000 m | 41.27 ± 12.9 | 62.86 ± 5.96 | 34.3 ± 2.7 | 67.89 ± 2.53 |
|  | 4 mm | | 1000 m | 49.79 ± 3.3 | 23.51 ± 1.65 | 58.4 ± 3.4 | 41.15 ± 5.60 |
|  |  |  | 2000 m | 54.90 ± 2.3 | 49.02 ± 5.76 | 46.2 ± 12.5 | 75.93 ± 8.39 |
|  |  |  | 3000 m | 33.83 ± 1.3 | 57.77 ± 5.91 | 37.0 ± 9.2 | 65.67 ­± 9.03 |

**SI 3.** Microbial biomass (C_mic_) and basal respiration (BR) in leaf and root litter in litterbags of 45 µm, 1 mm and 4 mm mesh exposed at 1000, 2000 and 3000 m for 6 and 12 months. Values are means ± SD.

|  | | | | **Leaf litter** | | **Root litter** | |
| --- | --- | --- | --- | --- | --- | --- | --- |
|  |  |  |  | **C_mic_**  **[mg C_mic_ g^-1^ dw]** | **BR**  **[μl O_2_ g^-1^ dw h^-1^]** | **C_mic_**  **[mg C_mic_ g^-1^ dw]** | **BR**  **[μl O_2_ g^-1^ dw h^-1^]** |
| **Time** | | | | | | | |
| 6 months | | | | 6.48 ± 1.29 | 74.46 ± 18.28 | 5.63 ± 1.43 | 52.18 ± 18.26 |
| 12 months | | | | 7.50 ± 1.41 | 94.31 ± 18.08 | 8.40 ± 2.66 | 104.33 ± 23.16 |
| **Mesh size** | | | | | | | |
| 45 µm | | | | 6.89 ± 1.03 | 92.71 ± 18.55 | 7.23 ± 3.07 | 77.21 ± 34.54 |
| 1 mm | | | | 6.22 ± 1.40 | 79.53 ± 20.68 | 6.76 ± 2.27 | 75.97 ± 31.12 |
| 4 mm | | | | 7.90 ± 1.36 | 80.91 ± 21.01 | 7.05 ± 2.31 | 81.58 ± 36.20 |
| **Altitude** | | | | | | | |
| 1000 m | | | | 7.01 ± 1.61 | 74.77 ± 18.99 | 9.36 ± 2.65 | 97.39 ± 33.65 |
| 2000 m | | | | 7.76 ± 1.06 | 94.39 ± 20.90 | 6.36 ± 1.37 | 73.18 ± 28.48 |
| 3000 m | | | | 6.22 ± 1.18 | 83.99 ± 17.96 | 5.31 ± 1.35 | 64.20 ± 30.37 |
| **Time × mesh size** | | | | | | | |
| 6 months | | 45 µm | | 6.37 ± 0.87 | 78.43 ± 9.49 | 6.03 ± 1.62 | 54.89 ± 22.10 |
|  |  | 1 mm | | 5.81 ± 1.25 | 66.60 ± 16.37 | 5.34 ± 1.57 | 51.04 ± 18.26 |
|  |  | 4 mm | | 7.28 ± 1.37 | 78.34 ± 24.96 | 5.50 ± 1.12 | 50.63 ± 15.81 |
| 12 months | | 45 µm | | 7.36 ± 0.97 | 106.98 ± 13.51 | 8.43 ± 3.76 | 99.54 ± 30.40 |
|  |  | 1 mm | | 6.63 ± 1.50 | 92.47 ± 16.25 | 8.18 ± 2.01 | 100.89 ± 18.05 |
|  |  | 4 mm | | 8.52 ± 1.09 | 83.48 ± 17.31 | 8.59 ± 2.18 | 112.54 ± 19.45 |
| **Time × altitude** | | | | | | | |
| 6 months | | 1000 m | | 5.90 ± 1.13 | 61.45 ± 13.44 | 7.07 ± 1.21 | 68.15 ± 17.25 |
|  |  | 2000 m | | 7.68 ± 1.11 | 89.29 ± 18.02 | 5.33 ± 0.97 | 50.35 ± 15.24 |
|  |  | 3000 m | | 5.88 ± 0.73 | 72.62 ± 11.77 | 4.49 ± 0.59 | 38.05 ± 6.22 |
| 12 months | | 1000 m | | 8.13 ± 1.21 | 88.10 ± 13.65 | 11.65 ± 1.26 | 126.62 ± 13.60 |
|  |  | 2000 m | | 7.84 ± 1.08 | 99.48 ± 23.35 | 7.40 ± 0.81 | 96.01 ± 17.87 |
|  |  | 3000 m | | 6. 55 ± 1.48 | 95.35 ± 16.01 | 6.14 ± 1.41 | 90.35 ± 19.55 |
| **Mesh size × altitude** | | | | | | | |
| 45 µm | | 1000 m | | 7.18 ± 0.89 | 88.15 ± 18.09 | 10.50 ± 2.96 | 109.46 ± 30.77 |
|  |  | 2000 m | | 7.34 ± 1.03 | 98.62 ± 20.28 | 6.42 ± 1.44 | 66.68 ± 27.52 |
|  |  | 3000 m | | 6.07 ± 0.77 | 91.35 ± 19.04 | 4.77 ± 0.54 | 55.50 ± 20.10 |
| 1 mm | | 1000 m | | 5.83 ± 1.51 | 67.66 ± 20.88 | 9.02 ± 1.90 | 95.16 ± 28.32 |
|  |  | 2000 m | | 7.49 ± 0.97 | 92.33 ± 23.60 | 5.77 ± 1.59 | 64.23 ± 23.55 |
|  |  | 3000 m | | 5.35 ± 0.68 | 78.62 ­± 9.70 | 5.49 ± 1.47 | 68.51 ± 35.51 |
| 4 mm | | 1000 m | | 8.04 ± 1.67 | 68.51 ± 11.83 | 8.56 ± 3.00 | 87.54 ± 42.46 |
|  |  | 2000 m | | 8.45 ± 0.99 | 92.21 ± 22.02 | 6.89 ± 1.04 | 88.63 ± 31.83 |
|  |  | 3000 m | | 7.22 ± 1.26 | 82.01 ± 23.15 | 5.69 ± 1.77 | 68.58 ± 36.52 |
| **Time × mesh size × Altitude** | | | | | | | |
| 6 months | 45 µm | | 1000 m | 6.62 ± 0.55 | 73.23 ± 9.53 | 7.93 ± 1.28 | 82.97 ± 3.69 |
|  |  |  | 2000 m | 6.98 ± 0.78 | 87.46 ± 6.52 | 5.26 ± 0.69 | 43.13 ± 10.54 |
|  |  |  | 3000 m | 5.52 ± 0.60 | 74.60 ± 6.51 | 4.90 ± 0.40 | 38.55 ± 6.15 |
|  | 1 mm | | 1000 m | 4.47 ± 0.29 | 48.82 ± 2.23 | 7.37 ± 0.16 | 71.59 ± 15.91 |
|  |  |  | 2000 m | 7.21 ± 0.25 | 72.29 ± 9.90 | 4.36 ± 0.18 | 43.37 ± 4.33 |
|  |  |  | 3000 m | 5.74 ± 0.71 | 78.69 ± 15.08 | 4.30 ± 0.70 | 38.15 ± 9.50 |
|  | 4 mm | | 1000 m | 6.61 ± 0.42 | 62.30 ± 13.34 | 5.89 ± 0.92 | 49.89 ± 8.71 |
|  |  |  | 2000 m | 8.85 ± 1.06 | 108.13 ± 13.68 | 6.35 ± 0.49 | 64.55 ± 18.58 |
|  |  |  | 3000 m | 6.39 ± 0.82 | 64.58 ± 11.24 | 4.26 ± 0.57 | 37.44 ± 5.05 |
| 12 months | 45 µm | | 1000 m | 7.75 ± 0.86 | 103.08 ± 7.71 | 13.06 ± 0.76 | 135.95 ± 15.77 |
|  |  |  | 2000 m | 7.71 ± 1.29 | 109.78 ± 24.73 | 7.59 ± 0.80 | 90.23 ± 10.89 |
|  |  |  | 3000 m | 6.63 ± 1.45 | 108.09 ± 4.75 | 4.64 ± 0.72 | 72.45 ± 10.52 |
|  | 1 mm | | 1000 m | 7.18 ± 0.40 | 86.49 ± 4.51 | 10.68 ± 0.93 | 118.72 ± 9.24 |
|  |  |  | 2000 m | 7.76 ± 1.44 | 112.37 ± 9.46 | 7.18 ± 0.55 | 85.09 ± 7.89 |
|  |  |  | 3000 m | 4.96 ± 0.44 | 78.54 ± 2.82 | 6.67 ± 0.84 | 98.87 ± 17.27 |
|  | 4 mm | | 1000 m | 9.46 ± 0.85 | 74.71 ± 7.50 | 11.23 ± 0.51 | 125.19 ± 13.35 |
|  |  |  | 2000 m | 8.04 ± 0.92 | 76.29 ± 16.28 | 7.43 ± 1.26 | 112.70 ± 21.19 |
|  |  |  | 3000 m | 8.06 ± 1.09 | 99.43 ± 17.40 | 7.11 ± 1.20 | 99.73 ± 19.98 |

**SI 4.** **Effect of altitude on (A) microbial biomass (C_mic_) and (B) basal respiration (BR) after 6 and 12 months.** Values are means ± SE. For each litter type, bars marked with different letters within each harvesting time differ significantly (Tukey’s HSD tests, p < 0.05).


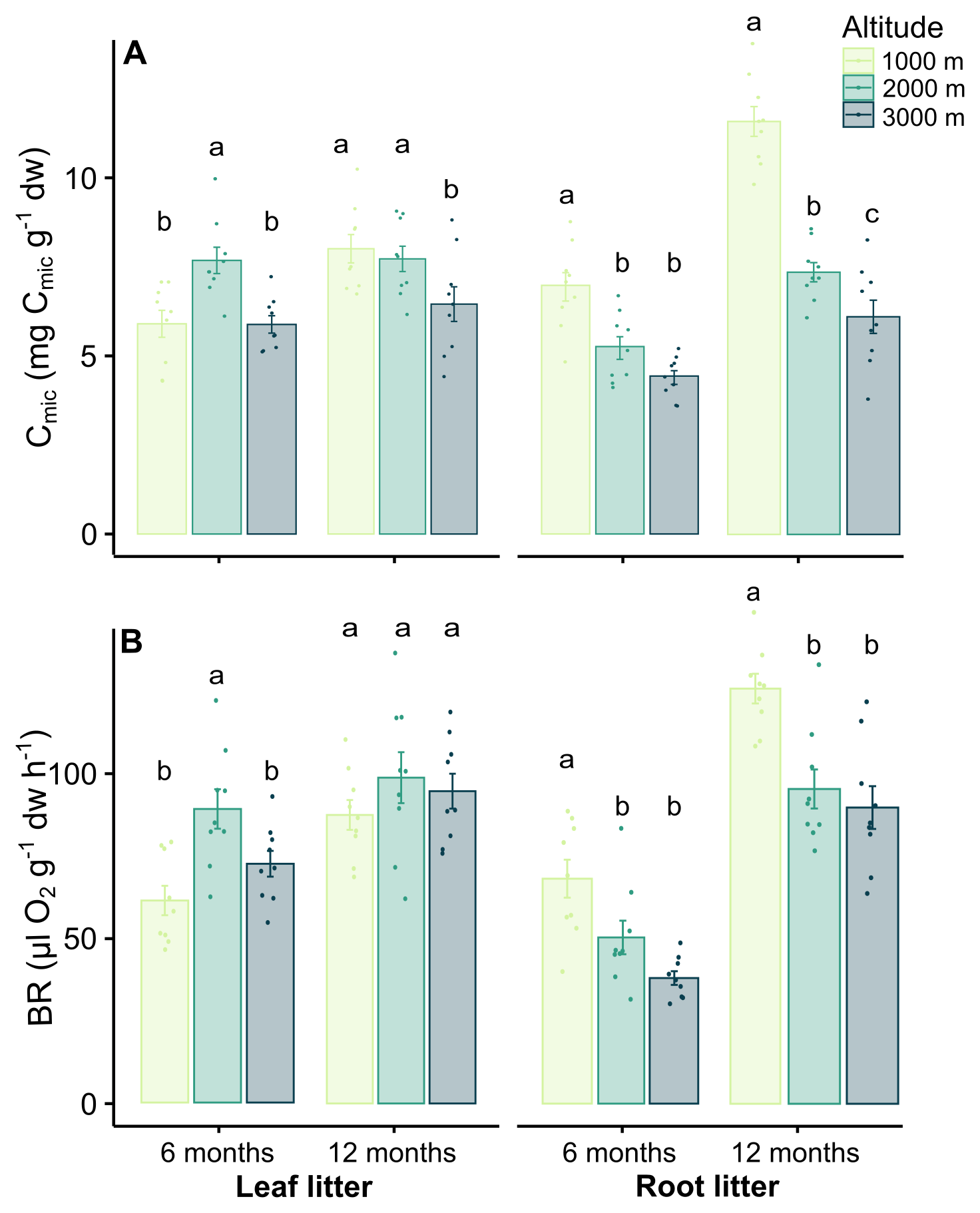


**SI 5.** Collembola and Oribatida abundance and Oribatida species richness in leaf and root litter in litterbags of 45 µm, 1 mm and 4 mm mesh exposed at 1000, 2000 and 3000 m for 6 and 12 months. Values are means ± SD.

|  | | | **Leaf litter** | | | **Root litter** | | |
| --- | --- | --- | --- | --- | --- | --- | --- | --- |
|  | | | **Abundance of Collembola**  **[ind. 10 g^−1^]** | **Abundance of Oribatida**  **[ind. 10 g^−1^]** | **Oribatida richness**  **[species 10 g^−1^]** | **Abundance of Collembola**  **[ind. 10 g^−1^]** | **Abundance of Oribatida**  **[ind. 10 g^−1^]** | **Oribatida richness**  **[species 10 g^−1^]** |
| **Time** | | | | | | | | |
| 6 months | | | 38 ± 49 | 37 ± 40 | 7 ± 8 | 65 ± 66 | 53 ± 70 | 11 ± 11 |
| 12 months | | | 24 ± 23 | 27 ± 28 | 6 ± 5 | 55 ± 51 | 37 ± 33 | 9 ± 7 |
| **Mesh size** | | | | | | | | |
| 45 µm | | | 28 ± 45 | 6 ± 9 | 2 ± 3 | 63 ± 61 | 24 ± 31 | 5 ± 5 |
| 1 mm | | | 34 ± 43 | 45 ± 38 | 9 ± 7 | 62 ± 65 | 50 ± 55 | 12 ± 8 |
| 4 mm | | | 32 ± 27 | 45 ± 34 | 7 ± 7 | 57 ± 52 | 61 ± 67 | 13 ± 10 |
| **Altitude** | | | | | | | | |
| 1000 m | | | 61 ± 40 | 42 ­± 37 | 11 ± 8 | 105 ± 55 | 98 ± 66 | 19 ± 9 |
| 2000 m | | | 24 ± 37 | 43 ± 38 | 7 ± 5 | 64 ± 53 | 20 ± 14 | 6 ± 3 |
| 3000 m | | | 8 ± 9 | 11 ± 13 | 2 ± 2 | 13 ± 14 | 17 ± 16 | 5 ± 4 |
| **Time × mesh size** | | | | | | | | |
| 6 months | 45 µm | | 41 ± 60 | 4 ± 6 | 1 ± 1 | 80 ± 76 | 24 ± 29 | 5 ± 5 |
|  | 1 mm | | 41 ± 56 | 58 ±44 | 11 ± 8 | 67 ± 80 | 58 ± 74 | 12 ± 10 |
|  | 4 mm | | 33 ± 30 | 49 ± 36 | 10 ± 8 | 48 ± 35 | 78 ± 88 | 15 ± 13 |
| 12 months | 45 µm | | 14 ± 15 | 9 ± 10 | 3 ± 4 | 45 ± 39 | 24 ± 35 | 5 ± 5 |
|  | 1 mm | | 26 ± 26 | 32 ± 27 | 8 ± 5 | 56 ± 49 | 42 ± 30 | 11 ± 7 |
|  | 4 mm | | 32 ± 26 | 42 ± 33 | 8 ± 6 | 65 ± 66 | 44 ± 32 | 11 ± 7 |
| **Time × altitude** | | | | | | | | |
| 6 months | 1000 m | | 80 ± 44 | 58 ± 46 | 13 ± 11 | 122 ±67 | 124 ­± 83 | 22 ± 11 |
|  | 2000 m | | 30 ± 49 | 44 ± 40 | 6 ± 4 | 66 ± 47 | 21 ± 13 | 6 ± 3 |
|  | 3000 m | | 4 ± 4 | 9 ± 7 | 2 ± 2 | 8 ± 11 | 15 ± 14 | 4 ± 3 |
| 12 months | 1000 m | | 42 ± 26 | 26 ± 16 | 9 ± 5 | 89 ± 36 | 72 ± 28 | 16 ± 6 |
|  | 2000 m | | 18 ± 20 | 43 ± 39 | 7 ± 6 | 62 ± 61 | 20 ± 16 | 6 ± 4 |
|  | 3000 m | | 13 ± 11 | 13 ± 17 | 2 ± 2 | 15 ± 16 | 19 ± 18 | 5 ± 5 |
| **Mesh size × altitude** | | | | | | | | |
| 45 µm | 1000 m | | 47 ± 41 | 10 ± 12 | 4 ± 4 | 116 ± 57 | 63 ± 22 | 12 ± 3 |
|  | 2000 m | | 29 ± 63 | 6 ± 7 | 2 ± 2 | 66 ± 47 | 8 ± 8 | 3 ± 1 |
|  | 3000 m | | 7 ± 13 | 2 ± 5 | 1 ± 1 | 7 ± 18 | 2 ± 2 | 1 ± 1 |
| 1 mm | 1000 m | | 76 ± 53 | 59 ± 35 | 15 ± 7 | 110 ± 72 | 100 ± 75 | 20 ± 10 |
|  | 2000 m | | 16 ± 7 | 67 ± 38 | 9 ± 4 | 62 ± 56 | 21 ± 9 | 7 ± 1 |
|  | 3000 m | | 8 ± 9 | 10 ± 6 | 3 ± 2 | 13 ± 12 | 29 ± 17 | 8 ­± 4 |
| 4 mm | 1000 m | | 60 ± 23 | 59 ± 39 | 15 ± 8 | 90 ± 37 | 132 ± 75 | 25 ± 8 |
|  | 2000 m | | 27 ± 20 | 57 ± 31 | 9 ± 3 | 64 ± 64 | 31 ± 14 | 9 ± 4 |
|  | 3000 m | | 10 ± 7 | 21 ± 18 | 3 ± 2 | 15 ± 11 | 20 ± 9 | 5 ± 2 |
| **Time × mesh size × Altitude** | | | | | | | | |
| 6 months | 45 µm | 1000 m | 68 ± 50 | 1 ± 1 | 0 ± 1 | 149 ± 59 | 61 ± 5 | 11 ± 4 |
|  |  | 2000 m | 55 ± 89 | 9 ± 9 | 2 ± 2 | 92 ± 52 | 11 ± 12 | 3 ± 2 |
|  |  | 3000 m | 0 ± 0 | 1 ± 1 | 0 ± 1 | 0 ± 0 | 1 ± 1 | 0 ± 1 |
|  | 1 mm | 1000 m | 101 ± 64 | 88 ± 19 | 20 ± 8 | 129 ± 103 | 128 ± 102 | 24 ± 11 |
|  |  | 2000 m | 17 ± 6 | 75 ± 50 | 8 ± 3 | 68 ± 61 | 23 ± 10 | 7 ± 2 |
|  |  | 3000 m | 5 ± 3 | 11 ± 5 | 4 ± 1 | 5 ± 3 | 23 ± 17 | 5 ± 2 |
|  | 4 mm | 1000 m | 72 ± 8 | 86 ± 28 | 19 ± 6 | 87 ± 21 | 184 ± 74 | 32 ± 6 |
|  |  | 2000 m | 18 ± 9 | 47 ± 24 | 8 ± 1 | 37 ± 9 | 28 ± 14 | 8 ± 4 |
|  |  | 3000 m | 8 ± 4 | 14 ± 6 | 3 ± 1 | 20 ± 14 | 21 ± 7 | 5 ± 2 |
| 12 months | 45 µm | 1000 m | 25 ± 16 | 19 ± 11 | 7 ± 4 | 82 ± 37 | 65 ± 34 | 12 ± 3 |
|  |  | 2000 m | 3 ± 3 | 3 ± 3 | 2 ± 2 | 39 ± 26 | 5 ± 3 | 3 ± 2 |
|  |  | 3000 m | 15 ± 16 | 4 ± 7 | 1 ± 2 | 15 ± 25 | 3 ± 3 | 1 ± 2 |
|  | 1 mm | 1000 m | 51 ± 32 | 29 ± 8 | 11 ± 2 | 92 ± 38 | 71 ± 33 | 17 ± 8 |
|  |  | 2000 m | 15 ± 9 | 59 ± 31 | 10 ± 5 | 55 ± 65 | 19 ± 8 | 6 ± 2 |
|  |  | 3000 m | 11 ± 12 | 9 ± 8 | 2 ± 2 | 21 ± 14 | 34 ± 19 | 10 ± 4 |
|  | 4 mm | 1000 m | 48 ± 30 | 31 ± 27 | 10 ± 8 | 93 ± 48 | 80 ± 24 | 19 ± 2 |
|  |  | 2000 m | 37 ± 25 | 66 ± 40 | 10 ± 5 | 91 ± 90 | 34 ± 17 | 9 ± 4 |
|  |  | 3000 m | 12 ± 11 | 27 ± 25 | 3 ± 3 | 10 ± 6 | 19 ± 13 | 5 ± 3 |

**SI 6. Effect of mesh size and altitude on Collembola and Oribatida abundance in leaf and root litter.** Variations in (A) Collembola and (B) Oribatida abundance in leaf and root litter in litterbags of different mesh size (45 µm, 1 mm and 4 mm) at three altitudes (1000, 2000 and 3000 m). Values are means ± SE. Bars marked with different letters within each altitude differ significantly (Tukey’s HSD tests, p < 0.05).

**
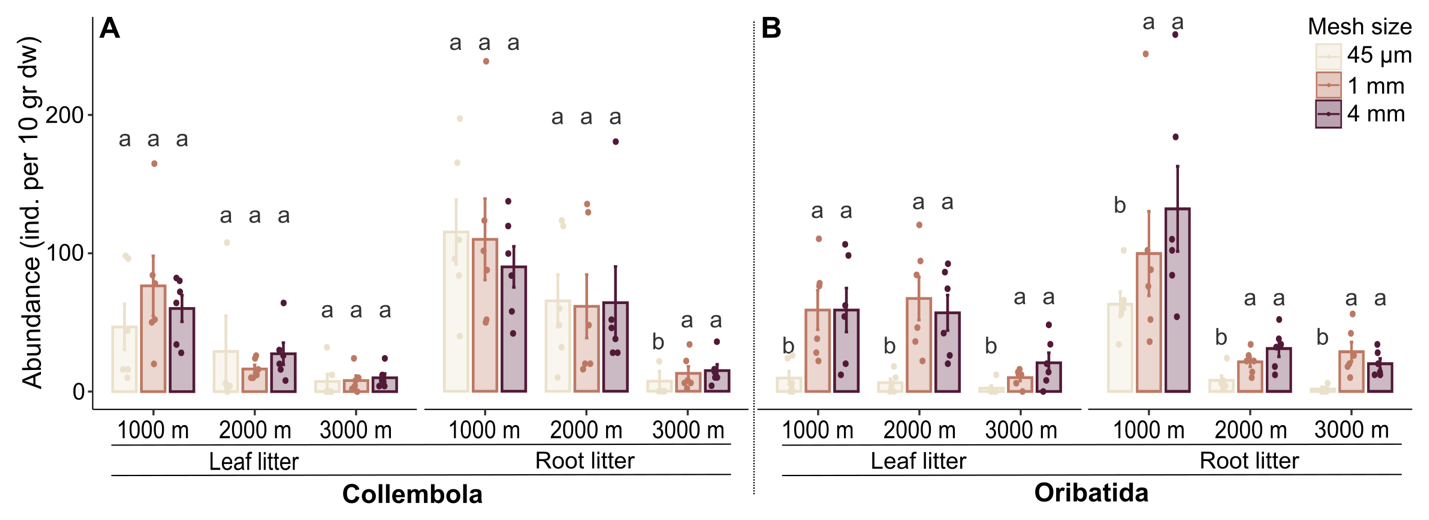
**

**SI 7.** List of Oribatida species, abbreviations used in Figure 5 and their abundance in leaf and root litter; n sp, presumed new species.

| **No.** | **Species** | **Abbreviation** | **Leaves** | **Included in CCA leaves** | **Abundance in leaves** | **Roots** | **Included in CCA roots** | **Abundance in roots** |
| --- | --- | --- | --- | --- | --- | --- | --- | --- |
| 1 | *Acrogalumna cubana* |  | x | - | 2 | - | - | 0 |
| 2 | *Acrogalumna* n. sp 1 | *AcrogSp1* | x | x | 6 | x | - | 2 |
| 3 | *Amazoppia tricuspidata* |  | - | - | 0 | x | - | 2 |
| 4 | *Arceremaeus incaensis* |  | x | - | 2 | - | - | 0 |
| 5 | *Arcoppia dechambrierorum* | *ArcpDech* | - | - | 0 | x | x | 3 |
| 6 | *Arcoppia tripartita* |  | - | - | 0 | x | - | 1 |
| 7 | *Beckiella elongata* |  | x | - | 1 | - | - | 0 |
| 8 | *Brachioppia deliciosa* | *BracDelc* | x | x | 6 | x | x | 13 |
| 9 | *Brachioppia* n. sp 2 |  | - | - | 0 | x | - | 1 |
| 10 | *Brachioppia* sp 3 | *BrachSp3* | x | - | 2 | x | x | 22 |
| 11 | *Brachioppiella* n. sp 2 | *BrachSp2* | - | - | 0 | x | x | 46 |
| 12 | *Campachipteria brevisetosa* |  | x | - | 1 | - | - | 0 |
| 13 | *Caudamaeolus petalus* | *CaudPetl* | - | - | 0 | x | x | 3 |
| 14 | *Ceratorchestes globosus* |  | x | - | 2 | - | - | 0 |
| 15 | *Ceratorchestes* sp 1 |  | x | - | 1 | - | - | 0 |
| 16 | *Ceratozetes* n. sp 1 |  | x | - | 1 | - | - | 0 |
| 17 | *Cosmozetes* n. sp 2 |  | x | - | 1 | - | - | 0 |
| 18 | *Cosmozetes* n. sp 3 |  | x | - | 1 | - | - | 0 |
| 19 | *Cosmozetes reticulatus* | *CosmRetc* | x | x | 35 | x | x | 37 |
| 20 | *Crotonia reticulata* |  | x | - | 1 | - | - | 0 |
| 21 | *Cultroribula zicsii* | *CultZics* | x | x | 7 | x | x | 6 |
| 22 | *Cyrthermannia florence* | *CyrtFlor* | - | - | 0 | x | x | 12 |
| 23 | *Damaeus flagellatus* | *DamaFlag* | x | x | 5 | x | - | 1 |
| 24 | *Dynatozetes* n. sp 1 |  | - | - | 0 | x | - | 2 |
| 25 | *Enarthronota* n. sp 1 |  | x | - | 1 | x | - | 2 |
| 26 | *Eohypochthonius gracilis* | *EohpGrac* | x | x | 5 | x | x | 39 |
| 27 | *Eohypochthonius* n. sp 1 | *EohypSp1* | x | x | 3 | x | x | 11 |
| 28 | *Epidamaeus meridianus* |  | - | - | 0 | x | - | 1 |
| 29 | *Epieremulus granulatus* | *EpieGran* | x | x | 7 | x | - | 1 |
| 30 | *Epieremulus longiseta* |  | x | - | 2 | - | - | 0 |
| 31 | *Epieremulus* n. sp 1 | *EpierSp1* | x | x | 10 | - | - | 0 |
| 32 | *Epilohmannia minuta minuta* | *EplMinMn* | - | - | 0 | x | x | 6 |
| 33 | *Eremobelba foliata* | *EremFoli* | - | - | 0 | x | x | 5 |
| 34 | *Eremulus brasiliensis* | *EremBras* | x | - | 3 | - | - | 0 |
| 35 | *Eremulus rigidisetosus* | *EremRigd* | x | x | 7 | x | x | 5 |
| 36 | *Fenestrobelba subcomplexa* | *FensSubc* | x | x | 8 | x | x | 6 |
| 37 | *Fosseremus laciniatus* | *FossLacn* | x | x | 3 | x | x | 8 |
| 38 | *Galumna flabellifera* | *GalmFlab* | x | x | 10 | x | x | 5 |
| 39 | *Galumna* n. sp 1 |  | - | - | 0 | x | - | 1 |
| 40 | *Gehypochthonius* n. sp 1 |  | x | - | 1 | x | - | 2 |
| 41 | *Gitella variabilis* | *GitlVari* | - | - | 0 | x | x | 6 |
| 42 | *Gittella maxima* |  | - | - | 0 | x | - | 2 |
| 43 | *Gittella minor* | *GittMinr* | - | - | 0 | x | x | 3 |
| 44 | *Gittella* n. sp 1 | *GittlSp1* | x | - | 1 | x | x | 9 |
| 45 | *Hammerella parasufflata* | *HammPars* | - | - | 0 | x | x | 5 |
| 46 | *Haplobelba* n. sp 1 |  | - | - | 0 | x | - | 1 |
| 47 | *Haplobelba simplex* | *HaplSimp* | x | x | 4 | x | x | 9 |
| 48 | *Haplozetes paraminimicoma* | *HaplParm* | x | - | 1 | x | x | 10 |
| 49 | *Hemileius hemileiformis* | *HemlHeml* | x | x | 18 | x | x | 6 |
| 50 | *Hemileius* n. sp 1 | *HemilSp1* | x | x | 28 | x | - | 2 |
| 51 | *Hemileius parvus* |  | x | - | 1 | x | - | 2 |
| 52 | *Heminothrus castaneus* |  | x | - | 1 | - | - | 0 |
| 53 | *Hermannobates bifurcatus* |  | x | - | 1 | x | - | 1 |
| 54 | *Hermannobates monstruosus* |  | - | - | 0 | x | - | 1 |
| 55 | *Hermannobates* n. sp 6 | *HermnSp6* | - | - | 0 | x | x | 3 |
| 56 | *Kokoppia dudichi* |  | x | - | 1 | x | - | 2 |
| 57 | *Kokoppia euramosa* |  | - | - | 0 | x | - | 2 |
| 58 | *Lanceoppia* n. sp 1 | *LanceSp1* | x | x | 3 | - | - | 0 |
| 59 | *Lanceoppia* n. sp 2 | *LanceSp2* | x | x | 6 | - | - | 0 |
| 60 | *Lanceoppia zicsica* | *LancZics* | x | x | 3 | - | - | 0 |
| 61 | *Lasiobelba chistyakovi* | *LasiChis* | x | - | 1 | x | x | 41 |
| 62 | *Lichochthonius mollis* |  | - | - | 0 | x | - | 1 |
| 63 | *Licnozetes granulatus* | *LicnGran* | x | x | 10 | x | x | 15 |
| 64 | *Liebstadia* n. sp 1 | *LiebsSp1* | x | x | 9 | x | x | 3 |
| 65 | *Liebstadia* n. sp 11 | *LiebSp.1a* | x | x | 5 | - | - | 0 |
| 66 | *Liebstadia* n. sp 2 |  | x | - | 2 | - | - | 0 |
| 67 | *Machadobelba* n. sp 1 | *MachdSp1* | - | - | 0 | x | x | 5 |
| 68 | *Malacoangelia remigera* | *MalcRemg* | - | - | 0 | x | x | 7 |
| 69 | *Malaconothrus monodactylus* | *MalcMond* | x | x | 5 | x | x | 5 |
| 70 | *Malaconothrus* n. sp 3 |  | x | - | 1 | x | - | 2 |
| 71 | *Malaconothrus* n. sp 4 |  | - | - | 0 | x | - | 2 |
| 72 | *Malaconothrus* n. sp 5 | *MalacSp5* | x | x | 6 | - | - | 0 |
| 73 | *Microtegeus borhidii* | *MicrBorh* | x | x | 12 | - | - | 0 |
| 74 | *Microtegeus similis* | *MicrSiml* | x | x | 5 | x | - | 1 |
| 75 | *Nanhermannia elegantissima* | *NanhEleg* | x | - | 2 | x | x | 12 |
| 76 | *Nanhermannia nana* form 1 | *NanhNana* | - | - | 0 | x | x | 13 |
| 77 | *Neoamerioppia longiclava* | *NeoaLong* | x | x | 132 | x | - | 1 |
| 78 | *Neoamerioppia longicoma* | *NeoaLogc* | x | x | 8 | - | - | 0 |
| 79 | *Neoamerioppia rotunda* | *NeoaRotn* | x | x | 24 | - | - | 0 |
| 80 | *Neoamrioppia espelatiarum* | *NeoaEspl* | x | x | 3 | - | - | 0 |
| 81 | *Neoctenogalumna longiciliata* |  | x | - | 1 | - | - | 0 |
| 82 | *Neosuctobelba transitoria* |  | x | - | 2 | - | - | 0 |
| 83 | *Opiella nova* | *OpieNova* | x | x | 27 | x | x | 36 |
| 84 | *Oribatella* n. sp 2 | *OribtSp2* | x | x | 10 | x | x | 4 |
| 85 | *Oripoda* n. sp 1 | *OripdSp1* | x | x | 3 | - | - | 0 |
| 86 | *Orthozetes bidentatus* | *OrthBidn* | x | - | 2 | x | x | 20 |
| 87 | *Oxyoppia polynesia* | *OxyoPoln* | x | x | 7 | - | - | 0 |
| 88 | *Parhypochthonius* n. sp 1 |  | x | - | 1 | - | - | 0 |
| 89 | *Pergalumna australis* |  | - | - | 0 | x | - | 1 |
| 90 | *Pergalumna sura* | *PergSura* | x | x | 4 | x | x | 5 |
| 91 | *Perscheloribates luminosus* |  | x | - | 2 | x | - | 2 |
| 92 | *Perscheloribates paratzitzikamaensis* |  | - | - | 0 | x | - | 2 |
| 93 | *Plenotocepheus neotropicus* | *PlenNeot* | x | - | 2 | x | x | 8 |
| 94 | *Protoribates ecuadoriensis* | *ProtEcua* | x | - | 2 | x | x | 13 |
| 95 | *Protoribates* n. sp 3 | *ProtrSp3* | x | x | 4 | x | x | 25 |
| 96 | *Protoribates* n. sp 4 |  | - | - | 0 | x | - | 1 |
| 97 | *Protoribates paracapucinus* | *ProtParc* | x | x | 8 | x | x | 29 |
| 98 | *Pulchroppia* n. sp 1 |  | x | - | 1 | - | - | 0 |
| 99 | *Ramusella puertomonttensis* | *RamsPuer* | - | - | 0 | x | x | 3 |
| 100 | *Rhynchoribates mirus* | *RhynMirs* | x | - | 2 | x | x | 7 |
| 101 | *Rhynchoribates* n. sp 1 |  | x | - | 1 | x | - | 1 |
| 102 | *Rioppia comteae* | *RiopComt* | x | - | 2 | x | x | 6 |
| 103 | *Rostrozetes carinatus* | *RostCarn* | x | x | 41 | x | - | 1 |
| 104 | *Rostrozetes glaber* |  | x | - | 1 | x | - | 1 |
| 105 | *Rostrozetes ovulum ovulum* | *RosOvlOv* | x | x | 11 | x | x | 39 |
| 106 | *Rostrozetes ovulum poensis* | *RosOvlPo* | x | x | 7 | x | x | 16 |
| 107 | *Rostrozetes* sp 1 | *RostrSp1* | x | - | 2 | x | x | 3 |
| 108 | *Rostrozetes* sp 3 | *RostrSp3* | x | - | 1 | x | x | 6 |
| 109 | *Rostrozetes* sp 4 | *RostrSp4* | x | x | 5 | x | x | 6 |
| 110 | *Rostrozetes* sp 5 | *RostrSp5* | x | - | 2 | x | x | 6 |
| 111 | *Rostrozetes* sp 6 | *RostrSp6* | x | x | 21 | x | x | 16 |
| 112 | *Scapheremaeus bicornutus* |  | - | - | 0 | x | - | 1 |
| 113 | *Scapheremaeus fungisetosus* |  | x | - | 2 | - | - | 0 |
| 114 | *Schalleria brevisetosa* | *SchlBrev* | - | - | 0 | x | x | 5 |
| 115 | *Schalleria pectinata* | *SchlPect* | - | - | 0 | x | x | 22 |
| 116 | *Scheloribates laticlava* | *SchlLatc* | x | - | 1 | x | x | 3 |
| 117 | *Scheloribates artigasi* |  | - | - | 0 | x | - | 1 |
| 118 | *Scheloribates diversidactylus* |  | x | - | 1 | - | - | 0 |
| 119 | *Scheloribates elegans* |  | x | - | 1 | - | - | 0 |
| 120 | *Scheloribates huancayensis* sp |  | - | - | 0 | x | - | 1 |
| 121 | *Scheloribates* n. sp 1 |  | x | - | 1 | x | - | 1 |
| 122 | *Scheloribates* n. sp 11 |  | - | - | 0 | x | - | 1 |
| 123 | *Sellnickochthonius elsosneadensis* sp | *SellElso* | x | x | 11 | - | - | 0 |
| 124 | *Sellnickochthonius muara* sp | *SellMuar* | - | - | 0 | x | x | 5 |
| 125 | *Sellnickochthonius tropic* |  | x | - | 2 | - | - | 0 |
| 126 | *Solenozetes carinatus* | *SolnCarn* | x | x | 6 | x | x | 39 |
| 127 | *Solenozetes flagellatus* | *SolnFlag* | x | x | 4 | x | x | 9 |
| 128 | *Solenozetes* n. sp 1 | *SolenSp1* | - | - | 0 | x | x | 52 |
| 129 | *Sternoppia brasiliensis* | *SterBras* | x | - | 2 | x | x | 3 |
| 130 | *Sternoppia fissurata* | *SterFiss* | - | - | 0 | x | x | 14 |
| 131 | *Sternoppia incisa* | *SterIncs* | x | - | 1 | x | x | 10 |
| 132 | *Sternoppia mirabilis* | *SterMirb* | x | x | 5 | - | - | 0 |
| 133 | *Sternoppia* n. sp 1 |  | - | - | 0 | x | - | 1 |
| 134 | *Sternoppia* n. sp 3 | *SternSp3* | - | - | 0 | x | x | 5 |
| 135 | *Sternoppia* n. sp 6 | *SternSp6* | - | - | 0 | x | x | 4 |
| 136 | *Sternoppia* n. sp 8 |  | - | - | 0 | x | - | 1 |
| 137 | *Sternoppia paraincisa* | *SterPara* | x | - | 1 | x | x | 22 |
| 138 | *Sternoppia paramirabilis* | *SterParm* | - | - | 0 | x | x | 5 |
| 139 | *Striatoppia opuntiseta* |  | - | - | 0 | x | - | 1 |
| 140 | *Striatoppia silvicola* |  | - | - | 0 | x | - | 1 |
| 141 | *Suctobelba compacta* |  | x | - | 1 | - | - | 0 |
| 142 | *Suctobelbella andrassyi* |  | - | - | 0 | x | - | 1 |
| 143 | *Suctobelbella baculifera* |  | - | - | 0 | x | - | 1 |
| 144 | *Suctobelbella claviseta* | *SuctClav* | x | x | 7 | x | x | 19 |
| 145 | *Suctobelbella complexa* | *SuctComp* | x | x | 16 | x | x | 4 |
| 146 | *Suctobelbella complexa* sp form 2 | *SucComF2* | x | x | 14 | x | x | 34 |
| 147 | *Suctobelbella loksai* | *SuctLoks* | x | - | 1 | x | x | 4 |
| 148 | *Suctobelbella macrodentata* |  | x | - | 1 | x | - | 2 |
| 149 | *Suctobelbella* n. sp 1 |  | - | - | 0 | x | - | 1 |
| 150 | *Suctobelbella* n. sp 3 | *SuctbSp3* | - | - | 0 | x | x | 3 |
| 151 | *Suctobelbella peracuta* | *SuctPerc* | x | - | 1 | x | x | 4 |
| 152 | *Suctobelbella perdentata* |  | x | - | 2 | x | - | 1 |
| 153 | *Suctobelbella semiplumosa indica* | *SucSemIn* | x | x | 5 | x | - | 2 |
| 154 | *Suctobelbella semiplumosa* sp form 1 | *SucSemF1* | x | x | 13 | x | x | 15 |
| 155 | *Suctobelbella variosetosa* |  | x | - | 2 | - | - | 0 |
| 156 | *Suctobelbila* n. sp 1 |  | - | - | 0 | x | - | 1 |
| 157 | *Suctobelbila* n. sp 2 |  | x | - | 1 | x | - | 1 |
| 158 | *Suctobelbila* n. sp 3 | *SuctbSp3* | - | - | 0 | x | - | 2 |
| 159 | *Suctobelbila* n. sp 5 |  | - | - | 0 | x | - | 1 |
| 160 | *Suctobelbila peruensis* | *SuctPeru* | x | x | 7 | x | - | 2 |
| 161 | *Suctoribates* n. sp 1 | *SuctrSp1* | x | x | 4 | x | x | 4 |
| 162 | *Suctoribates Oxyamerus n* |  | x | - | 1 | - | - | 0 |
| 163 | *Tecteremaeus cornutus* | *TectCorn* | x | - | 2 | x | x | 23 |
| 164 | *Tecteremaeus incompletus* | *TectIncm* | x | x | 4 | x | x | 13 |
| 165 | *Tectocepheus minor* | *TectMinr* | x | x | 5 | x | - | 1 |
| 166 | *Tectocepheus velatus sarekensis* | *TecVelSr* | x | x | 3 | - | - | 0 |
| 167 | *Tectocepheus velatus velatus* | *TecVelVl* | x | x | 3 | - | - | 0 |
| 168 | *Tegeocranellus bolivianus* | *TegeBolv* | x | x | 14 | x | x | 3 |
| 169 | *Teratoppia* n. sp 1 | *TeratSp1* | x | x | 3 | x | x | 5 |
| 170 | *Teratoppia pluripectinata* | *TertPlur* | x | x | 17 | x | x | 16 |
| 171 | *Xenillus* n. sp 2 |  | - | - | 0 | x | - | 1 |
| 172 | *Xenillus setiger* |  | - | - | 0 | x | - | 1 |
| 173 | *Xenolohmannia comosa* | *XenlComs* | x | - | 1 | x | x | 3 |
| 174 | *Yoshiobodes* n. sp 1 |  | - | - | 0 | x | - | 1 |
| 175 | *Yoshiobodes* n. sp 2 |  | - | - | 0 | x | - | 1 |
| 176 | *Zetomimus polpaicoensis* |  | x | - | 1 | - | - | 0 |
